# Intramyocardial angiogenetic stem cells and epicardial erythropoietin save the acute ischemic heart

**DOI:** 10.1242/dmm.033282

**Published:** 2018-06-22

**Authors:** Christian Klopsch, Anna Skorska, Marion Ludwig, Heiko Lemcke, Gabriela Maass, Ralf Gaebel, Martin Beyer, Cornelia Lux, Anita Toelk, Karina Müller, Christian Maschmeier, Sarah Rohde, Petra Mela, Brigitte Müller-Hilke, Stefan Jockenhoevel, Brigitte Vollmar, Robert Jaster, Robert David, Gustav Steinhoff

**Affiliations:** 1 Reference and Translation Center for Cardiac Stem Cell Therapy, Rostock University Medical Center, 18055 Rostock, Germany; 2 Department of Cardiac Surgery, Heart Center Rostock, University of Rostock, 18055 Rostock, Germany; 3 Division of Gastroenterology, Department of Medicine II, Rostock University Medical Center, 18055 Rostock, Germany; 4 Department of Tissue Engineering and Textile Implants, AME-Helmholtz Institute for Biomedical Engineering, RWTH Aachen University, 52074 Aachen, Germany; 5 Institute of Immunology & Core Facility for Cell Sorting and Cell Analysis, Rostock University Medical Center, 18055 Rostock, Germany; 6 Institute for Experimental Surgery, Rostock University Medical Center, 18055 Rostock, Germany

**Keywords:** Angiogenesis, Growth factor, Mesenchymal cardiac stem cells, Ischemia, Proliferation, Signaling

## Abstract

Ischemic heart failure is the leading cause of mortality worldwide. An early boost of intracardiac regenerative key mechanisms and angiogenetic niche signaling in cardiac mesenchymal stem cells (MSCs) could improve myocardial infarction (MI) healing. Epicardial erythropoietin (EPO; 300 U kg^−1^) was compared with intraperitoneal and intramyocardial EPO treatments after acute MI in rats (*n*=156). Real-time PCR and confocal microscopy revealed that epicardial EPO treatment enhanced levels of intracardiac regenerative key indicators (SDF-1, CXCR4, CD34, Bcl-2, cyclin D1, Cdc2 and MMP2), induced transforming growth factor β (TGF-β)/WNT signaling in intramyocardial MSC niches through the direct activation of AKT and upregulation of upstream signals FOS and Fzd7, and augmented intracardiac mesenchymal proliferation 24 h after MI. Cardiac catheterization and tissue analysis showed superior cardiac functions, beneficial remodeling and increased capillary density 6 weeks after MI. Concomitant fluorescence-activated cell sorting, co-cultures with neonatal cardiomyocytes, angiogenesis assays, ELISA, western blotting and RAMAN spectroscopy demonstrated that EPO could promote cardiomyogenic differentiation that was specific of tissue origin and enhance paracrine angiogenetic activity in cardiac CD45^−^CD44^+^DDR2^+^ MSCs. Epicardial EPO delivery might be the optimal route for efficient upregulation of regenerative key signals after acute MI. Early EPO-mediated stimulation of mesenchymal proliferation, synergistic angiogenesis with cardiac MSCs and direct induction of TGF-β/WNT signaling in intramyocardial cardiac MSCs could initiate an accelerated healing process that enhances cardiac recovery.

## Abstract (precis)

**Summary:** Cardiac mesenchymal stem cells respond early to myocardial infarction. Their regenerative capacity is directly upregulated by optimized stimulation of the heart through epicardial erythropoietin delivery.

## INTRODUCTION

Ischemic heart failure is the leading cause of death in the modern world. Further insight into the cell-specific mechanisms in specific cardiac stem cell niches of the adult ischemic heart could help us to understand their key roles in triggering regenerative impulses, reverse remodeling and restoring pump function. Previous studies underlined the importance of the epithelial-to-mesenchymal transition (EMT) mechanisms in epicardial stem cells and cardiac mesenchymal stem cell (MSC) niches during the initial phase after myocardial infarction (MI) (van Wijk et al., 2012; Chong et al., 2011). Recently, we demonstrated that the cardiac CD45^−^CD44^+^DDR2^+^ MSC subtype could take part in the very early cascades of myocardial regeneration (Klopsch et al., 2017). Synergistic interactions of erythropoietin (EPO) have been described with endothelial progenitor cells (EPCs) and with MSCs (Prunier et al., 2007; George et al., 2005; Copland et al., 2008). We, and others, have confirmed that EPO activates regeneration after MI, although dosing, spatial distribution and timing of EPO therapy might be crucial (Prunier et al., 2007; Moon et al., 2005; Lipsic et al., 2004; Klopsch et al., 2009; Gäbel et al., 2009; Kobayashi et al., 2008; Wang et al., 2009). Here, we show that epicardial EPO delivery could efficiently trigger early regenerative signaling and proliferation in the heart, induce direct transforming growth factor β (TGF-β)/WNT signaling in intramyocardial MSC niches and enhance angiogenesis, thus preserving cardiac function after acute MI. We provide the first evidence for an angiogenetic synergism of EPO with paracrine factors from post-ischemic cardiac CD45^−^CD44^+^DDR2^+^ MSCs and have investigated EPO-promoted cardiomyogenic differentiation in these cardiac MSCs. In addition, we have analyzed the human relevance of our findings in translational experiments.

## RESULTS

### Improved cardiac functions, remodeling and capillary density

**Fig. 1.**
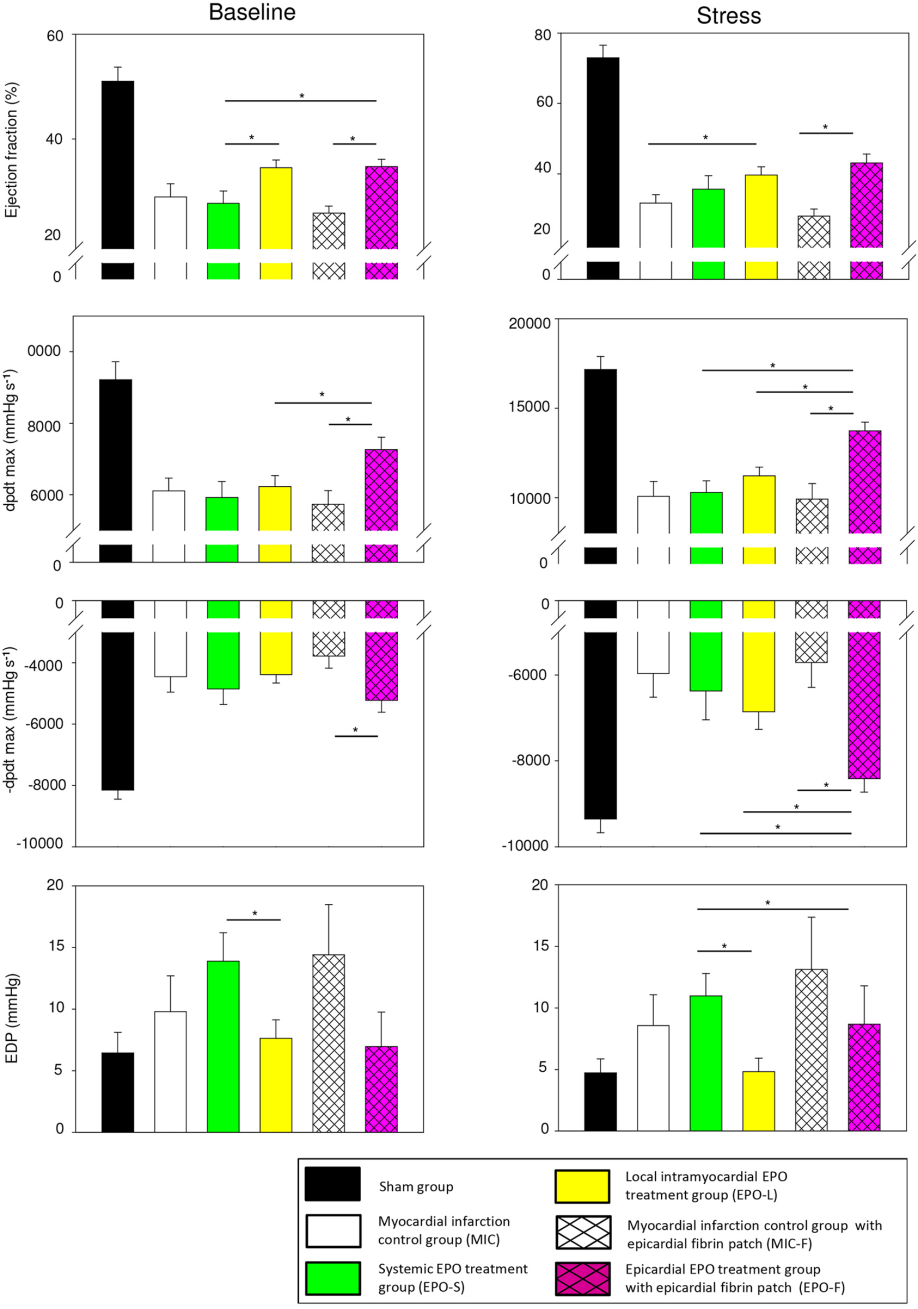
**Epicardial EPO delivery most efficiently restored cardiac functions 6 weeks after MI.** Cardiac functions under baseline (left panel) and dobutamine-induced (10 µg kg^−1^ min^−1^) stress conditions (right panel). dpdt indicates peak rate of maximum pressure rise (dpdt max) and decline (–dpdt max); EDP means end diastolic pressure. Sham group *n*=9; myocardial infarction control group (MIC) *n*=8; systemic EPO treatment group (EPO-S) *n*=9; local intramyocardial EPO treatment group (EPO-L) *n*=9; myocardial infarction control group with epicardial fibrin patch (MIC-F) *n*=6; epicardial EPO treatment group with epicardial fibrin patch (EPO-F) *n*=6. *P<0.05. Mean±s.e.m. See also Tables 1 and 2*.*

Six weeks after MI, the ejection fraction, contractility index peak rate of pressure rise (dpdt max) and relaxation index peak rate of pressure decline (–dpdt max) were increased by 15%, 38% and 47% after epicardial EPO delivery (group EPO-F) compared with a myocardial infarction control group with an epicardial fibrin patch (MIC-F; *P*<0.001, *P*=0.003 and *P*=0.002), respectively. Moreover, systolic and diastolic indices in EPO-F were significantly improved compared with a systemic EPO treatment group (EPO-S) and a local intramyocardial EPO treatment group (EPO-L; all *P*<0.05). Ejection fraction in EPO-L was moderately enhanced (by 8%) compared with a myocardial infarction control group (MIC; *P*=0.033), when there was no change in EPO-S (Fig. 1, Table 1 and Table 2; for detailed group definition see also Materials and Methods).

**Table 1.**
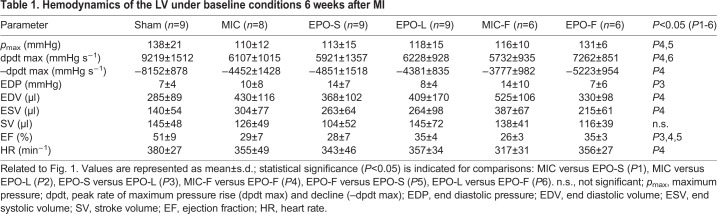
**Hemodynamics of the LV under baseline conditions 6 weeks after MI**

**Table 2.**
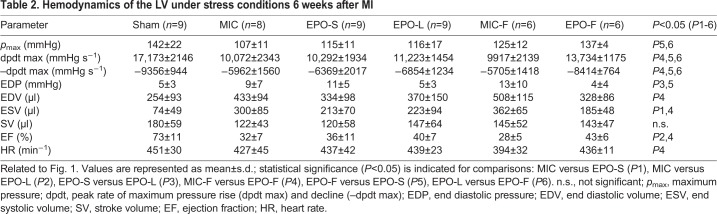
**Hemodynamics of the LV under stress conditions 6 weeks after MI**

**Fig. 2.**
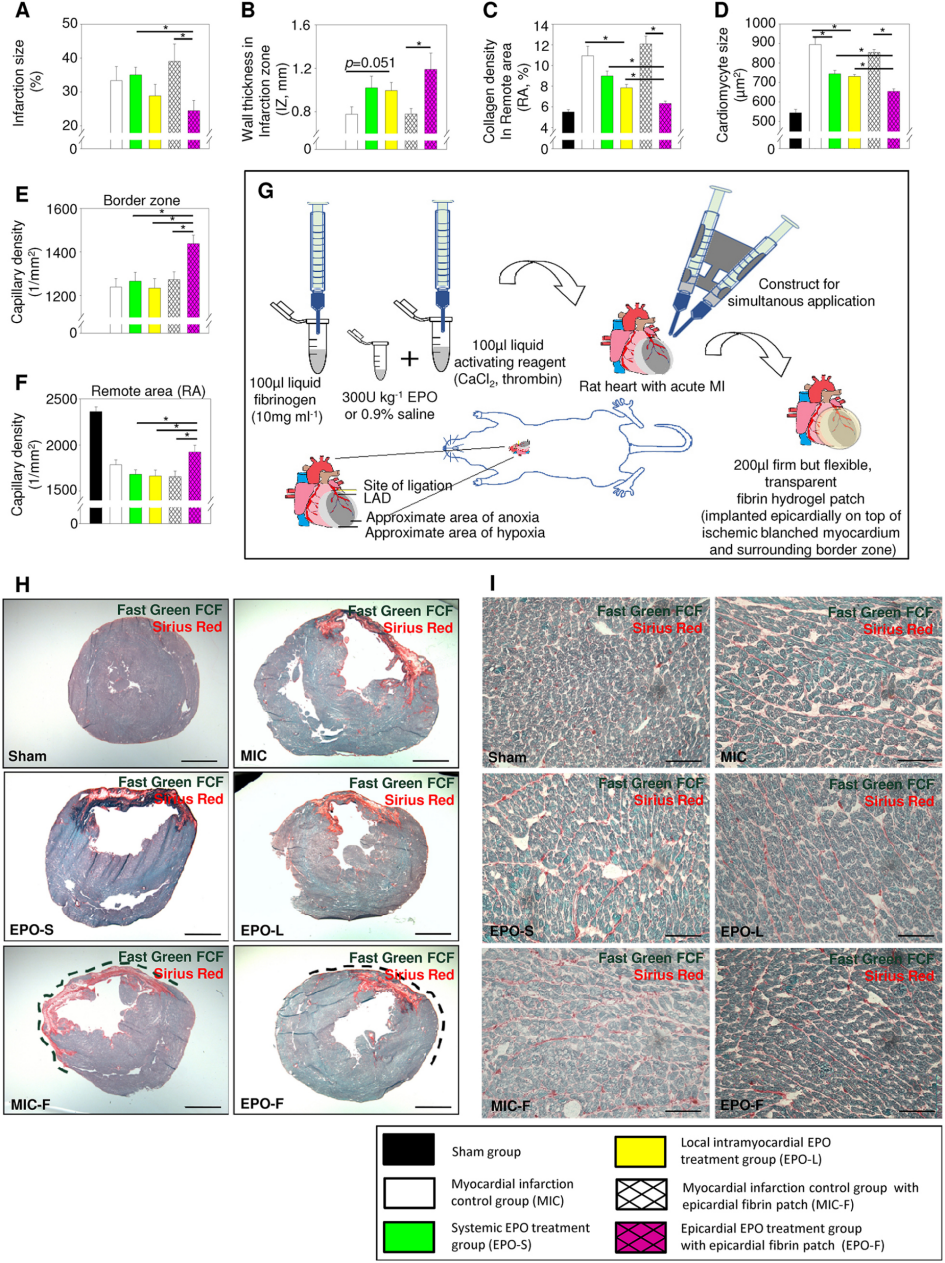
**Epicardial EPO delivery reduced infarction size, limited cardiac remodeling and enhanced capillary density 6 weeks after MI.** (A) Left ventricular infarction size (% of circumference) was decreased in EPO-F compared with MIC-F and EPO-S. (B) Left ventricular wall thinning at infarction zone (IZ) was limited in EPO-F compared with MIC-F. (C) Collagen density and (D) cardiomyocyte size in remote area (RA) of EPO-F hearts were at the lowest level compared with hearts from other groups that received MI. Capillary density was augmented in both (E) the border zone and (F) the remote area of EPO-F hearts at 6 weeks after MI compared with hearts from other groups that received MI. (G) Schematic indicating generation, dimension and characteristics of the applied epicardial fibrin patch on infarcted hearts. (H,I) Representative cross-sections of the heart (H; scale bars: 2 mm) and the RA of hearts (I; scale bars: 100 µm) stained with Sirius Red (red, fibrosis) and Fast Green FCF (green, myocytes) in Sham, MIC, EPO-S, EPO-L, MIC-F and EPO-F. The initial presence of the epicardial fibrin patch after MI (H, dashed line) was indicated in representative cross-sections of MIC-F and EPO-F hearts. *P<0.05; *n*=6 per group. Mean±s.e.m.

The infarction size in EPO-F was decreased significantly (by 14%) compared with MIC-F (*P*=0.031). Wall thickness at infarction zone (IZ) increased by 53% in EPO-F compared with MIC-F (*P*=0.040). Collagen density and cardiomyocyte size in the remote area (RA) were significantly decreased in EPO-F compared with MIC-F, EPO-S and EPO-L (all *P*<0.01). Capillary density significantly increased in EPO-F in the peri-infarction border zone (*P*=0.003, *P*=0.003 and *P*=0.050) and RA (*P*=0.007, *P*=0.012 and *P*=0.009) compared with MIC-F, EPO-S and EPO-L, respectively. It is noteworthy that collagen density in EPO-L and cardiomyocyte size in EPO-S and EPO-L were significantly reduced compared with MIC (*P*=0.011, *P*<0.001 and *P*<0.001, respectively; Fig. 2).

### Immediate EPO delivery, elevated tissue level and boosted intracardiac regeneration

**Fig. 3.**
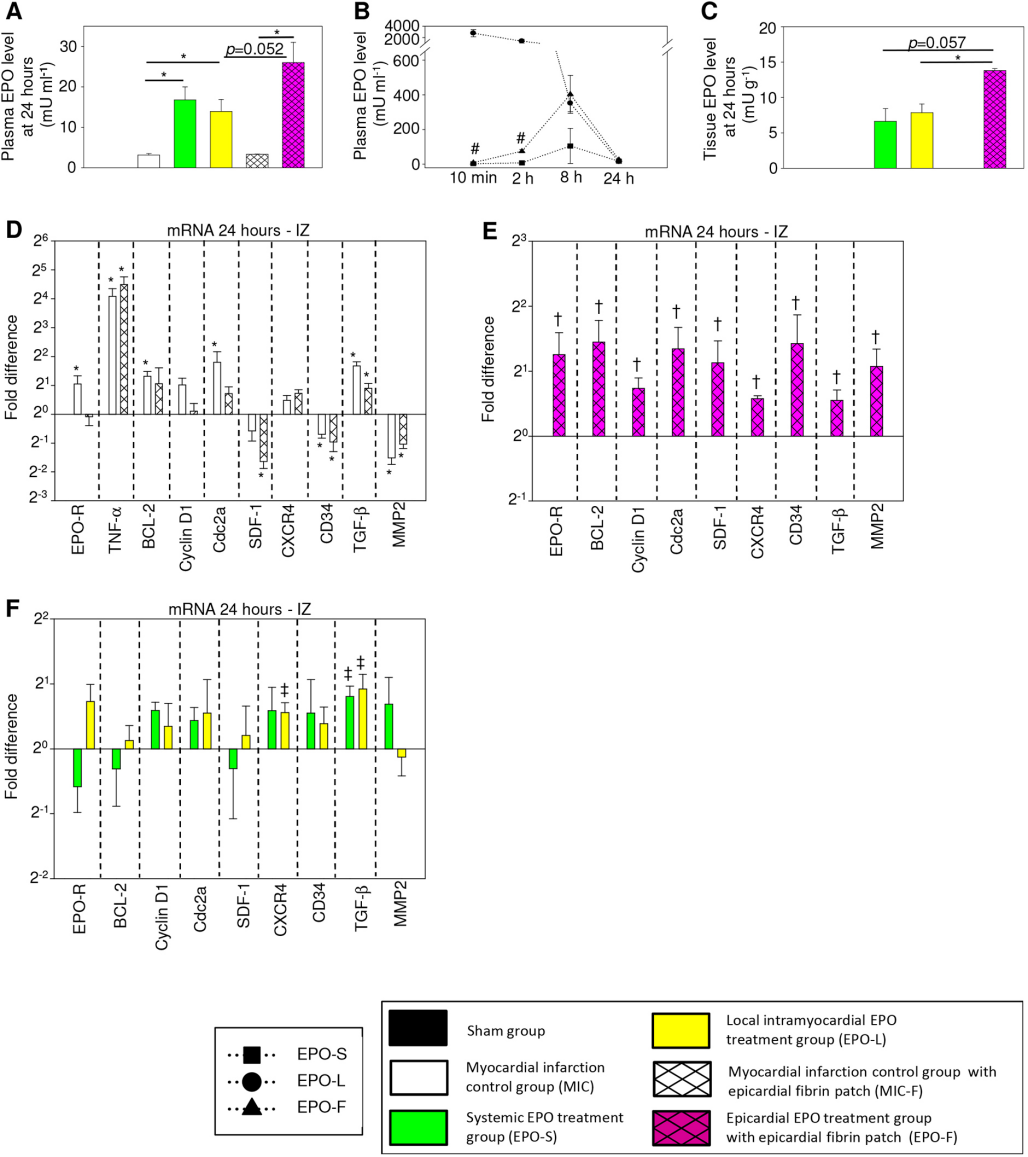
**Epicardial EPO delivery illustrated immediate EPO release, local drug retention and prompt intracardiac induction of numerous regenerative key factors.** (A-C) Plasma EPO level analyses depicted adequate EPO exposition (A) as well as immediate release and different systemic distribution kinetics (B) in all therapy groups at 10 min, 2 h, 8 h and 24 h after EPO administration. Intracardiac tissue EPO level (C) in the left ventricular septum was highest in EPO-F compared with EPO-L and EPO-S at 24 h after administration; *n*=7 in EPO-F, *n*=8 in other groups. *P<0.05. ^#^*P*<0.05 versus EPO-S and EPO-L. (D) Changes in gene expression of screened key factors caused by MI. Quantitative real-time PCR analysis for *Epo-R*, *Tnf-a*, *Sdf-1*, *Cxcr4*, *Cd34*, *Mmp2*, *Bcl-2*, cyclin D1 and *Cdc2* genes in infarction zone (IZ) of MIC and MIC-F compared with Sham hearts. The average mRNA expression level in the Sham hearts was arbitrarily given a value of 1 (2°); *P<0.05 versus Sham; *n*=6 per group. (E,F) Quantitative real-time PCR analysis for the same genes in IZ showed induction of all selected regenerative key factors in EPO-F compared with MIC-F hearts (E) and only a few changes in EPO-S and EPO-L compared with MIC hearts (F) at 24 h after MI. The average mRNA expression levels from MIC-F hearts (E) and MIC hearts (F) were arbitrarily given a value of 1 (2°). None of the EPO treatments changed the TNF-α gene expression level compared with their respective controls (data not shown); *n*=6 per group. ^†^*P*<0.05 versus MIC-F. ^‡^*P*<0.05 versus MIC. Mean±s.e.m.

Pharmacokinetic analysis revealed adequate EPO exposition in all therapy groups during the first 24 h after MI. In EPO-F, plasma levels were 314-fold and 18-fold lower compared with EPO-L (*P*=0.012, *P*=0.020), but 3-fold and 10-fold higher compared with EPO-S (*P*=0.028, *P*=0.005) at 10 min and 2 h after application, respectively. Relative reticulocyte count in EPO-F (3.4±0.6%) did not change compared with MIC-F (3.6±1.2%, *P*=0.637), but was increased by 0.9% and 0.8% in EPO-S and EPO-L compared with MIC (*P*=0.008 and *P*=0.006, respectively) at 24 h after MI. Remarkably, intracardiac EPO levels were elevated by 1.8-fold and 2.1-fold at 24 h in EPO-F compared with EPO-L and EPO-S (*P*=0.032 and *P*=0.057, respectively; Fig. 3A-C). Intracardiac EPO concentration in the myocardium of non-infarcted hearts (Sham group), MIC and MIC-F was not detectable in the applied set-up.

Real-time PCR analyses revealed a clear effect of MI on selected genes inside IZ at 24 h after MI. Inflammation marker tumor necrosis factor α (*Tnf-a*) showed mRNA elevations of 18.4-fold and 24.3-fold in groups MIC and MIC-F compared with Sham, respectively (all *P*<0.001). Other MI-related changes in gene expression levels uncovered partially limited comparability between groups MIC and MIC-F (see also Statistical analyses section). None of the EPO treatments additionally changed the *Tnf-a* gene expression level induced by MI (data not shown). However, all screened regenerative key factors were promptly induced inside IZ at 24 h after epicardial EPO delivery compared with MIC-F. Real-time PCR analyses in EPO-F illustrated mRNA elevations of EPO receptor (*Epo-R*) (2.8-fold, *P*<0.05), anti-apoptotic B-cell lymphoma 2 (*Bcl-2*, 3.1-fold, *P*=0.043), cell-cycle progression factor cyclin D1 (1.7-fold, *P*=0.034), cell division cycle 2 kinase (*Cdc2*, 2.9-fold, *P*=0.026), stem cell recruitment factor stromal cell-derived factor 1 (*Sdf-1*, 2.5-fold, *P*=0.041), SDF-1 receptor *Cxcr4* (1.5-fold, *P*=0.002), EPC surface marker *Cd34* (3.4-fold, *P*=0.027), *Tgf-b* (1.5-fold, *P*=0.043) and matrix metalloproteinase 2 (*Mmp2*, 2.2-fold, *P*=0.016) compared with MIC-F. It is of note that the intracardiac *Tgf-b* gene in EPO-L and EPO-S, as well as the *Cxcr4* gene in EPO-L, were induced compared with MIC (all *P*<0.05; Fig. 3D-F).

### Increased proliferation in intracardiac mesenchyme and triggered TGF-β/WNT signaling in cardiac MSC clusters early after MI

**Fig. 4.**
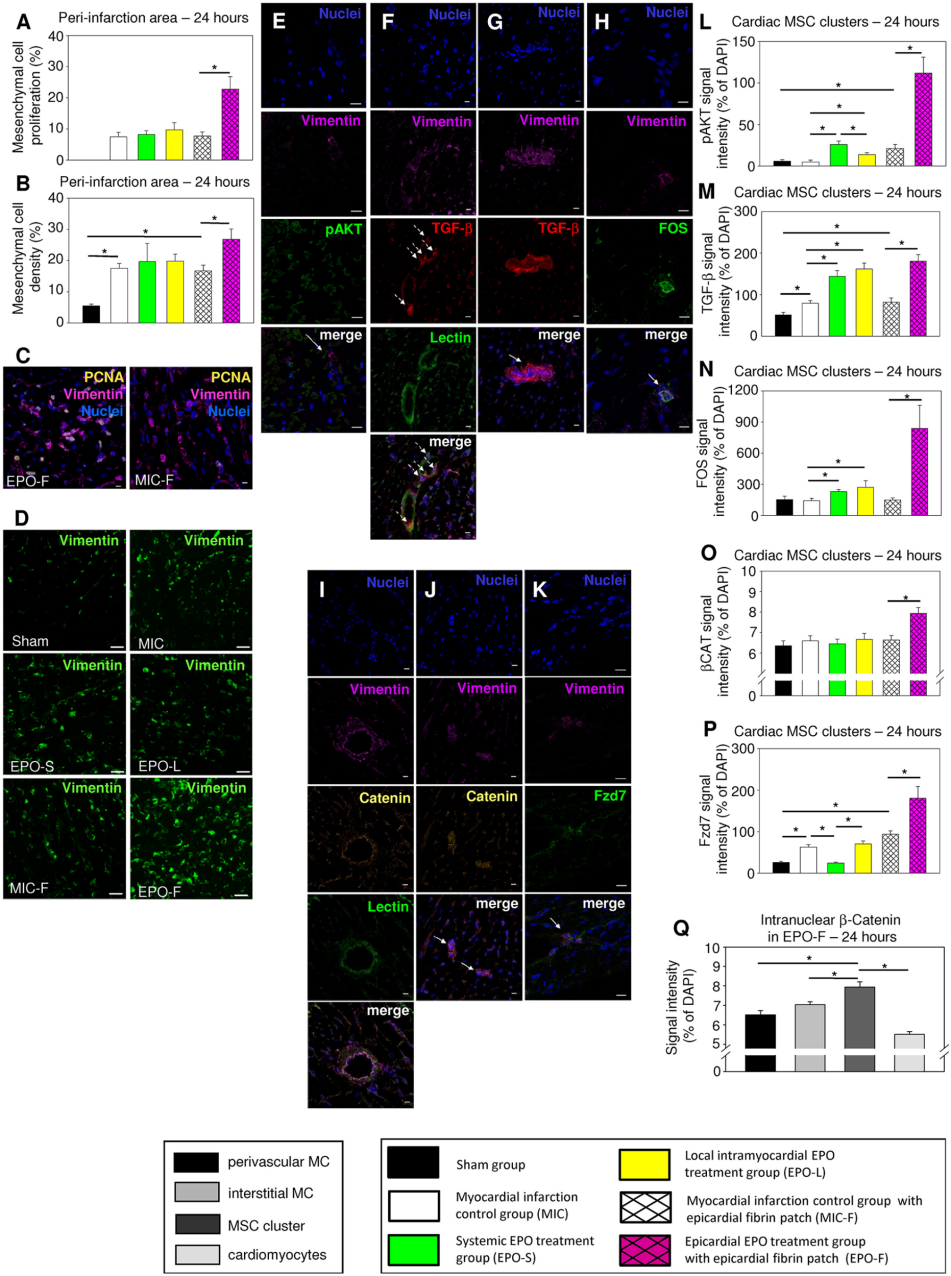
**Cardiac post-ischemic mesenchyme revealed increased proliferation hosting MSC clusters with induced TGF-β expression and WNT signaling early after epicardial EPO delivery.** (A,B) Proliferation in vimentin-positive mesenchymal cells (A) and intramyocardial mesenchymal cell density (B) were significantly higher in EPO-F than in MIC-F in the peri-infarction area at 24 h after MI. *P<0.05; *n*=6 per group. (C) Representative immunostaining images showing increased proliferation (PCNA, yellow) in vimentin-positive (magenta) mesenchymal cells in EPO-F compared with MIC-F in the peri-infarction area. Scale bars: 10 µm. (D) Representative immunostaining images for vimentin-positive (green) mesenchymal cell density in Sham as well as in the peri-infarction area of MIC, EPO-S, EPO-L, MIC-F and EPO-F. Scale bars: 100 µm. (E-K) Representative immunostaining images showing (E) phosphorylated AKT (pAKT, green), (F,G) TGF-β (red), (H) FOS (green), (I,J) β-catenin (yellow) and (K) Frizzled 7 (Fzd7, green) expression in vimentin-positive (magenta) intramyocardial MSC clusters (solid arrows; Klopsch et al., 2017) of EPO-F hearts at 24 h after MI. TGF-β signal was apparent in lectin-positive (green) perivascular vimentin-positive mesenchymal cells (F, dashed arrows) and reached maximal expression in intramyocardial MSC clusters (G, solid arrows). Here, TGF-β signal especially accented the margin of MSC clusters. The β-catenin signal was detected at intercellular adherens junctions throughout the myocardium (I). Scale bars: 10 μm. Blue, 4′,6-diamidino-2-phenylindole (DAPI) in nuclei. (L-Q) Quantified signal intensities for pAKT, TGF-β, FOS, intranuclear β-catenin and Fzd7 in cardiac MSC clusters in the peri-infarction area at 24 h after MI. Relative signal intensities (%) were calculated by normalization with fluorescence signal intensity. Specific to EPO-F (Q), intranuclear β-catenin signal intensity was clearly increased in MSC clusters (solid arrows) compared with perivascular mesenchymal cells (MC), interstitial MC and cardiomyocytes. *P<0.05. Mean±s.e.m.

Proliferation was predominately located intramyocardially and epicardially in the peri-infarction area. In vimentin-positive mesenchymal cells the proliferation was most dynamic and mesenchymal cell density augmented in the peri-infarction area of all MI groups at 24 h after MI. Importantly, the overall number of proliferating mesenchymal cells and the mesenchymal cell density in the peri-infarction area were further increased by 2.9-fold and 1.6-fold in EPO-F compared with MIC-F at 24 h after MI, respectively (*P*=0.005, *P*=0.027; Fig. 4A-D).

EPO-R downstream signal mediator protein kinase B (AKT) was activated in all EPO therapy groups in the peri-infarction area that hosted intramyocardial cardiac MSC clusters recently described by our laboratory (Klopsch et al., 2017). Focal analyses in the cardiac MSC clusters revealed that the signal intensity of phosphorylated AKT (pAKT) was augmented by 5.4-fold and 2.8-fold in the EPO therapy groups EPO-S and EPO-L compared with MIC and amplified by 5.3-fold in EPO-F compared with MIC-F at 24 h after MI (all *P*<0.001). Interestingly, the epicardial fibrin patch implantation alone without EPO treatment also resulted in AKT activation after MI in cardiac MSC clusters compared with the Sham group (3.7-fold; *P*=0.008). In addition, systemic intraperitoneal EPO treatment revealed greater pAKT signal intensity in cardiac MSC clusters than local intramyocardial EPO injections (1.9-fold; *P*=0.013).

The TGF-β signal expression was increased in perivascular mesenchymal cells and more apparently inside cardiac MSC clusters in the peri-infarction area and RA at 24 h after MI. Focal analyses revealed that TGF-β signal intensity was induced 1.6-fold in both MIC and MIC-F in cardiac MSC clusters in the peri-infarction area compared with Sham (*P*=0.005, *P*=0.013). The TGF-β signal in cardiac MSC clusters was further increased in the EPO therapy groups EPO-S and EPO-L compared with MIC and in EPO-F compared with MIC-F (1.8-fold, 2.0-fold, 2.2-fold; all *P*<0.01). Upstream signal mediator FOS was 1.6-fold, 1.9-fold and, remarkably, 5.6-fold augmented in cardiac MSC clusters in EPO therapy groups EPO-S, EPO-L and EPO-F compared with their respective controls (all *P*<0.001).

In addition, focal analyses in cardiac MSC clusters in the peri-infarction area revealed activated WNT signaling exclusively in EPO-F. The intranuclear signal intensity of β-catenin in intramyocardial MSC clusters was increased 20% in EPO-F compared with cardiac MSC clusters in MIC-F (*P*<0.001) and demonstrated relevant elevation compared with perivascular mesenchymal cells (*P*=0.007), interstitial mesenchymal cells (*P*=0.035) and cardiomyocytes specific to group EPO-F (*P*<0.001). WNT receptor Frizzled 7 (Fzd7), a promoter of WNT signaling, showed 2.4-fold and 3.6-fold increased signal intensities in cardiac MSC clusters in MIC and MIC-F compared with Sham, respectively (all *P*<0.001). Epicardial EPO treatment in EPO-F further increased the Fzd7 signal intensity 1.9-fold in cardiac MSC clusters compared with MIC-F (*P*=0.001). By contrast, systemic EPO therapy in EPO-S reduced the Fzd7 signal intensity in cardiac MSC clusters in our study (*P*<0.001, Fig. 4E-Q).

### EPO-mediated cardiomyogenic differentiation in rat cardiac CD45^−^CD44^+^DDR2^+^ MSCs

**Fig. 5.**
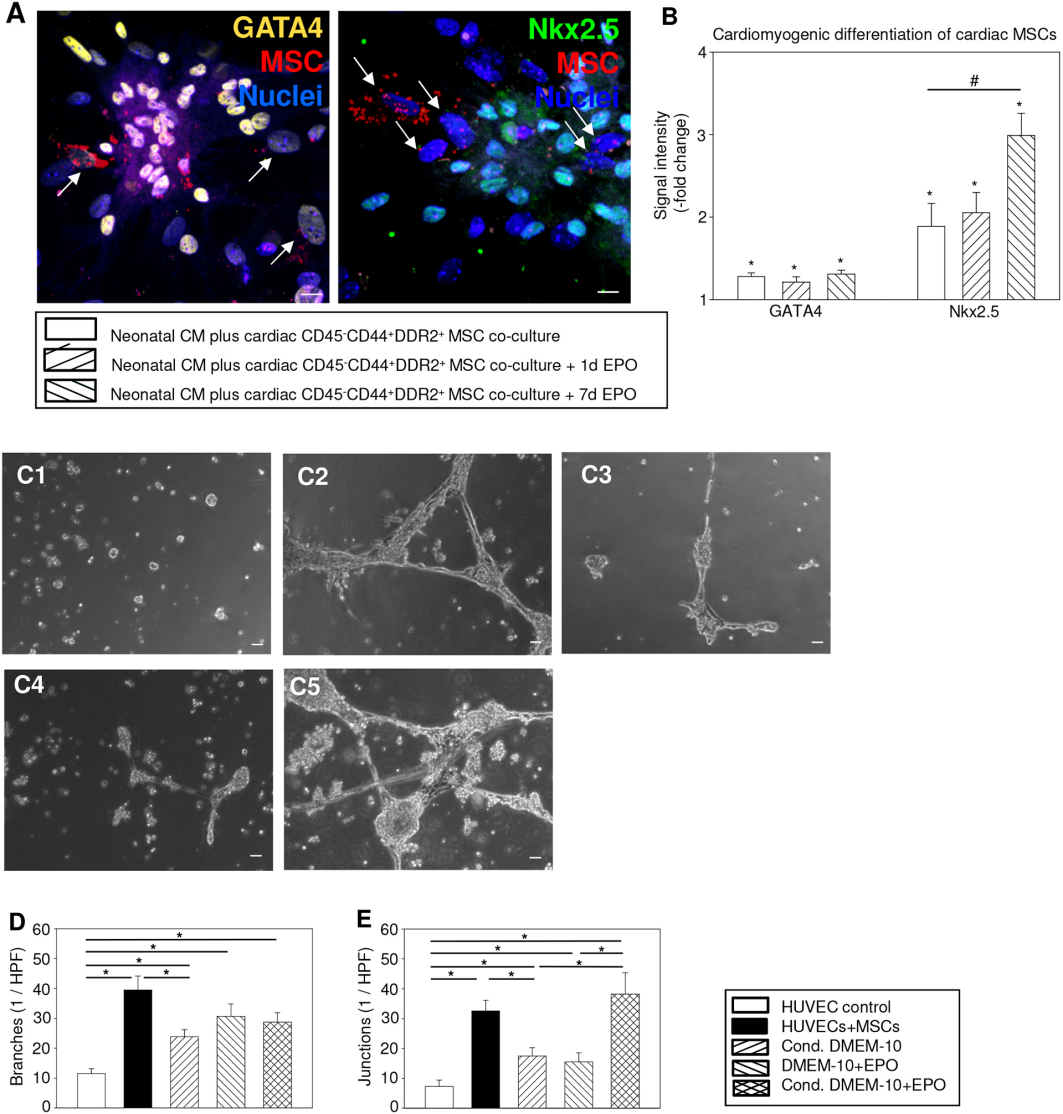
**EPO induced synergistic angiogenesis with rat cardiac CD45^−^CD44^+^DDR2^+^ MSCs and facilitated cardiomyogenic differentiation.** (A,B) Representative immunostaining images (A) from co-cultures of Qtracker^®^-labeled (red) cardiac CD45^−^CD44^+^DDR2^+^ MSCs and cardiomyocytes (CM). MSCs (arrows) show partially positive expression for cardiomyogenic transcription factors GATA4 (yellow) and Nkx2.5 (green). Quantified GATA4 and Nkx2.5 signal intensities (B) illustrated promotion of cardiomyogenic differentiation in co-cultured MSCs compared with mono-cultured MSCs and strongest Nkx2.5 signal expression in co-cultured MSCs by continuous EPO stimulation. The average signal intensities from MSCs in mono-culture were arbitrarily given a value of 1 (2°). *P<0.05 versus cardiac CD45^−^CD44^+^DDR2^+^ MSC mono-culture. ^#^*P*<0.05. Scale bars: 10 µm. Blue, DAPI in nuclei. (C-E) Representative phase-contrast microscopy images (C) from HUVECs in untreated mono-culture (C1, HUVEC control), in co-culture with rat cardiac CD45^−^CD44^+^DDR2^+^ MSCs (C2, HUVECs+MSCs), in MSC-conditioned medium (C3, Cond. DMEM-10), in EPO-supplemented medium (C4, DMEM-10+EPO) and in EPO-supplemented MSC-conditioned medium (C5, Cond. DMEM-10+EPO) with clear tubuli and network formations. Quantified HUVEC branching (D) and junctional network formation (E) illustrated synergistic angiogenetic potential of EPO and paracrine factors from cardiac CD45^−^CD44^+^DDR2^+^ MSCs. *P<0.05. Scale bars: 100 µm. Mean±s.e.m.

Rat cardiac CD45^−^CD44^+^DDR2^+^ MSCs provided the first evidence for EPO-mediated promotion of cardiomyogenic differentiation. Cardiac MSCs in all co-cultures with neonatal cardiomyocytes revealed increased expression of cardiomyogenic transcription factors Nkx2.5 and GATA-binding protein 4 (GATA4) compared with MSC mono-culture after 7 days. Moreover, continuous EPO stimulation augmented the Nkx2.5 signal intensity by 1.6-fold in co-cultured MSCs compared with the unstimulated co-culture (Fig. 5A,B, all *P*<0.05).

### Synergistic angiogenesis by EPO and rat cardiac CD45^−^CD44^+^DDR2^+^ MSCs

Following 24 h co-culture with post-ischemic CD45^−^CD44^+^DDR2^+^ MSCs, human umbilical cord vein endothelial cells (HUVECs) demonstrated significant angiogenesis. HUVECs showed clear tubuli and network formations. Branching and junctional network formation increased by 3.4-fold and 4.5-fold, respectively, compared with barely noticeable angiogenesis in unstimulated HUVEC mono-culture (both *P*<0.001). In the remaining stimulated HUVEC mono-culture groups, angiogenesis was activated. In-depth analyses showed improved cellular branching and junctional network formations especially in the HUVEC mono-culture supplemented with EPO and MSC-conditioned medium (Cond. DMEM-10+EPO). MSC-conditioned medium (Cond. DMEM-10) and EPO-supplemented medium (DMEM-10+EPO) showed similar angiogenesis induction on HUVEC branching and junctional network formation compared with unstimulated HUVEC mono-culture (all *P*<0.001). EPO supplementation in MSC-conditioned medium (Cond. DMEM-10+EPO) synergistically induced angiogenesis to a greater extent with clear tubuli and network formations: HUVEC branching increased 2.5-fold compared with unstimulated HUVEC mono-culture (*P*<0.001; Fig. 5C-E). In MSC-conditioned medium supplemented with EPO, HUVEC junctional network formation was augmented 5.2-fold (*P*<0.001), 2.2-fold (*P*=0.017) and 2.5-fold (*P*=0.007) compared with HUVEC mono-culture, Cond. DMEM-10 and DMEM-10+EPO, respectively (Fig. 5C-E).

### Human MSCs showed EPO-boosted TGF-β/WNT signaling and fibroblast differentiation

**Fig. 6.**
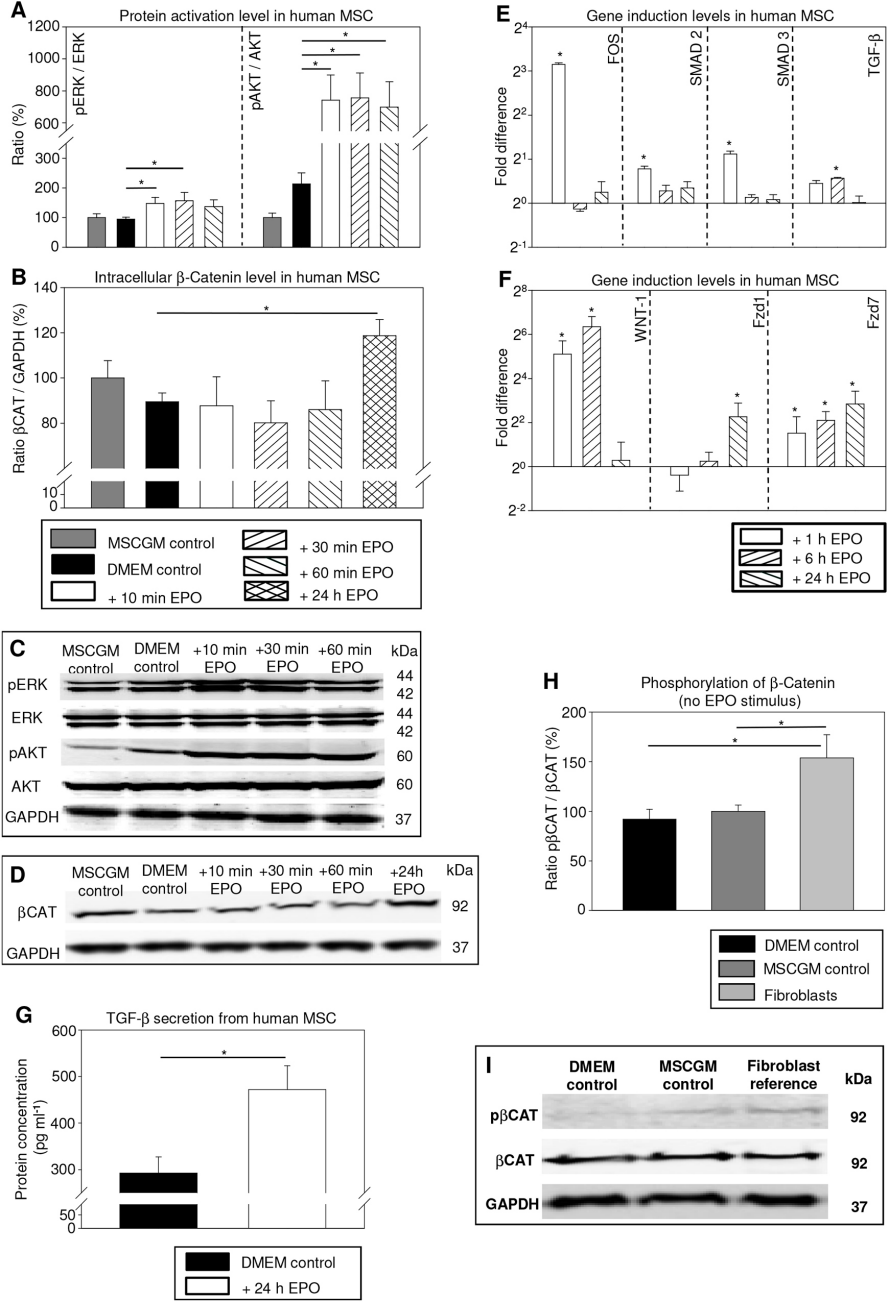
**Human MSCs revealed EPO activated AKT, the ERK/FOS/TGF-β axis, TGF-β secretion and WNT signaling in MSCs.** (A-D) Western blotting for the ratios pERK/ERK and pAKT/AKT (A) as well as for the ratio β-catenin/GAPDH (B) in MSCs. Expression was quantified and compared between the MSCGM control group, DMEM control group and DMEM+EPO (100 U ml^−1^) group regarding different EPO incubation times (10 min, 30 min, 60 min, 24 h). The averaged protein ratio was arbitrarily given a value of 100% for MSCGM control group. p means phosphorylated; βCAT indicates β-catenin; *n*=6 per group. *P<0.05. Gel bands (C,D) are shown for representative images. (E,F) Quantitative real-time PCR analysis for (E) *FOS*, *SMAD2*, *SMAD3* and *TGF-B1* as well as (F) *WNT-1*, *FZD1* and *FZD7* gene expression in MSCs. The average mRNA expression level was arbitrarily given a value of 1 (2°) for the DMEM control group. The mRNA expression levels were compared between DMEM control group and DMEM+EPO (100 U ml^−1^) group regarding different EPO incubation times (1 h, 6 h, 24 h); *n*=4 per group. *P<0.05 versus DMEM control group. (G) TGF-β secretion from MSCs was significantly higher in the DMEM+EPO group than in DMEM control group after 24 h EPO incubation; *n*=6 per group. *P<0.05. (H,I) Western blotting for the ratio pβCAT/βCAT in MSCs and fibroblasts. Expression was quantified (H) and compared between MSCs in the MSCGM control group, MSCs in the DMEM control group and fibroblasts. The averaged protein ratio was arbitrarily given a value of 100% for MSCGM control group; *n*=4 per group. *P<0.05. (I) Gel bands are shown for representative images. Mean±s.e.m.

*In vitro* analyses revealed a 1.6-fold higher extracellular signal-regulated kinase (ERK, *P*=0.032) and a 3.5-fold higher AKT (*P*=0.002) activation in human MSCs as early as 10 min after EPO stimulation compared with the group DMEM control. The ERK target gene *FOS* and TGF-β signaling mediators *SMAD2* and *SMAD3* were enhanced by 8.9-fold (*P*=0.029), 1.7-fold (*P*<0.001) and 2.2-fold (*P*<0.001) in MSCs, respectively, following 1 h of EPO incubation. With time delay, *TGF-B1* gene expression was significantly induced by 1.5-fold (*P*=0.029) in MSCs at 6 h after EPO stimulation. Finally, TGF-β secretion by MSCs was 61% elevated after 24 h of EPO stimulation (*P*=0.020). In parallel, after 24 h of EPO incubation the intracellular β-catenin content in MSCs was significantly increased by 29.2% compared with the DMEM control group (*P*=0.007). Gene expression for WNT-promoting ligand *WNT-1* was dramatically induced by 67.8-fold (*P*=0.002) and 109.7-fold (*P*=0.002) at 1 h and 6 h after starting EPO conditioning, respectively. Concordantly, the mRNA levels of WNT receptors were also increased for *FZD1* (24 h, 8.6-fold, *P*=0.010) and for *FZD7* (1 h, 8.4-fold, *P*=0.021; 6 h, 5.4-fold, *P*<0.001; 24 h, 12.0-fold, *P*<0.001; Fig. 6A-G) after EPO stimulation.

Interestingly, WNT signaling inhibition (presented as the ratio of intracellular phosphorylated β-catenin to entire β-catenin content) was 61.9% and 53.9% lower in MSCs in the DMEM control and standard mesenchymal stem cell growth medium (MSCGM) control groups compared with vascular fibroblasts, respectively (*P*=0.041 and *P*=0.049; Fig. 6H,I). The entire β-catenin content did not significantly differ between these groups (C.K. and R.J., unpublished).

**Fig. 7.**
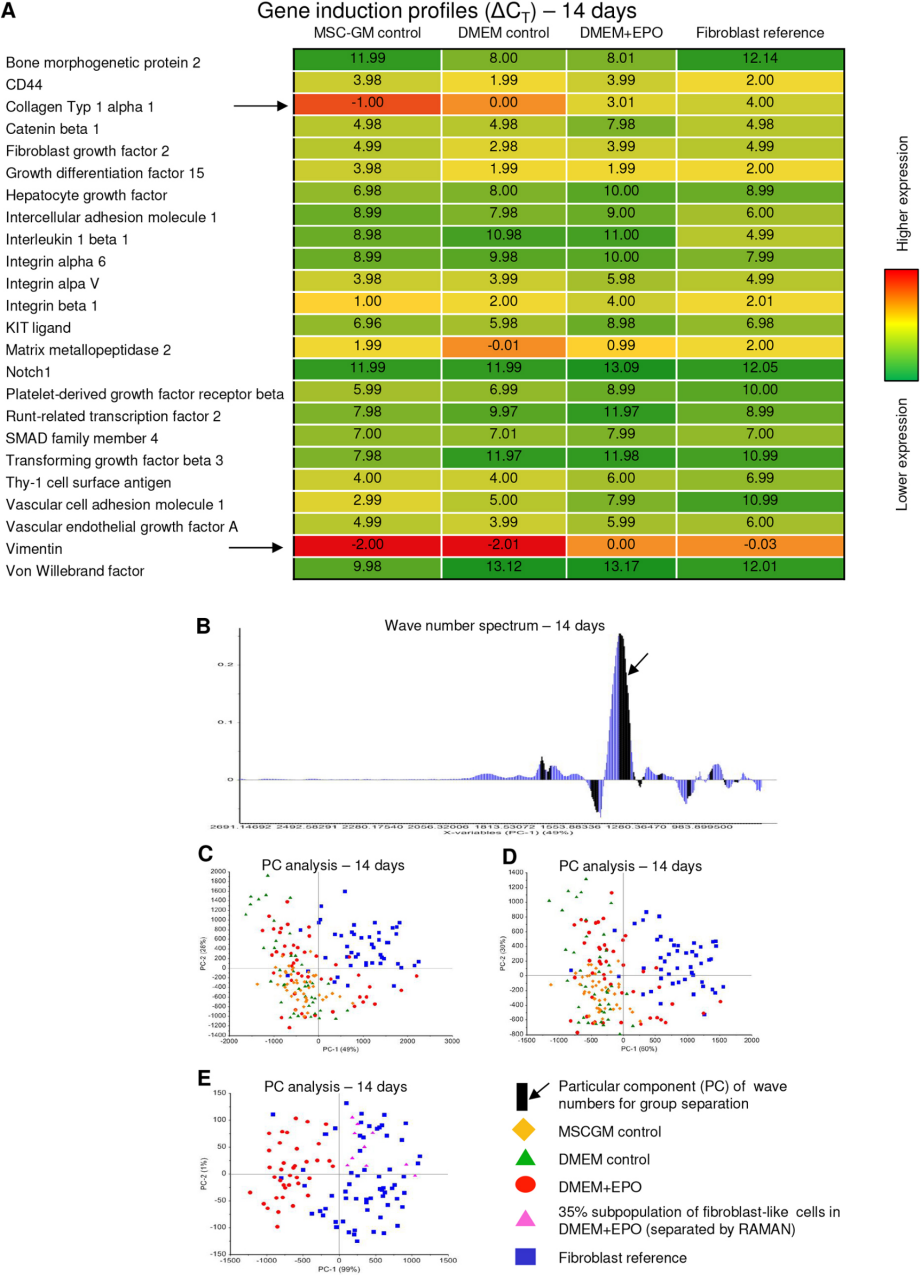
**Human bone-marrow-derived MSCs revealed partial fibroblastic differentiation facilitated by EPO.** (A) Heat map for quantitative real-time PCR array data depicted similar gene expression for collagen 1α1 and vimentin (arrows) in fibroblasts and continuously EPO-treated MSCs after 14 days of cultivation. ΔCT values were calculated for MSCs in MSCGM control group, MSCs in the DMEM control group, MSCs in DMEM+EPO (100 U ml^−1^) group and fibroblasts. (B-E) RAMAN spectroscopy applied the particular component (PC, arrow) analysis along the entire wave number spectrum of all loaded cells (B) for group separation (C) after 14 days of continuous cultivation. Separation along wave numbers characteristic for collagen (D,E) revealed fibroblasts were clearly different from MSCs in control groups, whereas an approximate 35% subpopulation of EPO-treated MSCs had differentiated into a fibroblast-like cell subpopulation (E).

The mRNA profiling indicated that gene expression for collagen 1α1 and vimentin in fibroblasts and continuously long-term EPO-treated MSCs were similarly low compared with untreated MSCs. Using advanced RAMAN spectroscopy, applied fibroblasts could clearly be separated from untreated MSC controls. Importantly, we reproducibly enabled the separation of an approximate 35% subpopulation of EPO-treated MSCs that had developed collagen characteristics different from untreated MSCs, but similar to those of fibroblasts (99% separation relevance at the particular component of wave numbers 1329-1365 cm^−1^). This subpopulation of EPO-treated MSCs was not separable from applied fibroblasts (Fig. 7).

## DISCUSSION

Our results describe improved MI healing as a result of early intracardiac induction of EPO-mediated pleiotropic regenerative key processes. We demonstrate that epicardial EPO delivery not only induces early proliferation factors cyclin D1 and Cdc2, but also significantly increases dynamic proliferation in the early post-ischemic cardiac mesenchyme that hosts EPO-responsive highly angiogenetic cardiac MSCs. In a recent study from our laboratory, these intramyocardial MSC niches showed strong proliferation and the isolated cardiac CD45^−^CD44^+^DDR2^+^ MSC subtype illustrated typical phenotypic, immunogenic and functional MSC characteristics (Klopsch et al., 2017). The intracardiac Bcl-2 and TGF-β induction might have further supported early cardiac cell survival following epicardial EPO delivery (Parsa et al., 2003; Yang et al., 2012). Like others, we indicate that EPO-mediated cell protection and cardiac regeneration might be dependent on local EPO release kinetics and tissue concentrations (Wang et al., 2009).

Our previous studies and those of others have shown that the SDF-1/CXCR4 axis constitutes a particularly important ligand-receptor pair in EPO-mediated stem-cell mobilization and recruitment to the infarcted myocardium (Klopsch et al., 2009; Brunner et al., 2009): SDF-1 gradients can attract CXCR4^+^ hematopoietic stem cells, EPCs, MSCs and resident cardiac stem cells to the ischemic myocardium (Schuh et al., 2012; Zhuang et al., 2009; Tang et al., 2011). In our study, EPO induced the expression of TGF-β in the heart, which could have improved MSC recruitment through the SDF-1/CXCR4 axis (Si et al., 2014). Hoch and coworkers demonstrated that EPO is essential for angiogenesis mediated by cardiac progenitor cells (Hoch et al., 2011). Other studies reported that EPC mobilization seems dependent on EPO dosing and myocardial restoration is probably associated with both adequate EPC mobilization and significant SDF-1 induction in the ischemic heart (Prunier et al., 2007; Westenbrink et al., 2008; Allukian et al., 2013). Our results provide the first evidence that there might be a dependency on epicardial delivery and/or intramyocardial dose for the early EPO-mediated induction of the SDF-1/CXCR4 axis, the increased expression of EPC marker CD34, the enrichment of the early post-ischemic cardiac mesenchyme with highly angiogenetic cardiac MSCs and the enhanced angiogenesis in the ischemic heart.

One possible source for the early enriched post-ischemic mesenchyme might be proliferating resident cardiac MSC niches. Following MI, oxygen tension and infiltrating paracrine-active macrophages could be major regulators controlling the MSC niche (Freytes et al., 2013; Mohyeldin et al., 2010). Moreover, MSC proliferative, migratory and angiogenetic/vasculogenetic capacity might be gradable dependent on EPO dosing (Zwezdaryk et al., 2007; Ward and Stewart, 2008). Importantly, our results demonstrated that epicardially delivered EPO clearly increased TGF-β expression and WNT signaling in the proliferating intramyocardial MSC clusters already at day 1 after MI. Both TGF-β and WNT signaling are known to be key triggers of the EMT in stem cells, which mainly activates changes in transcription program, phenotype and migratory capacity (Gonzalez and Medici, 2014; Nishita et al., 2000). Chong et al. reported that resident cardiac MSCs predominately originate from the epicardium (Chong et al., 2011). Van Wijk et al. emphasized the early induction of EMT at the epicardium and reinduction of embryonic Wilms' Tumor 1 (*Wt1*) gene in proliferating and migrating epicardium-derived mesenchymal cells that were primarily detected at day 3 following MI. Interestingly, the study depicted only partial Wt1 lineage commitment in the proliferating mesenchymal cell compartment of the intramyocardial infarction border zone (van Wijk et al., 2012). We did not observe co-expression of proliferating cell nuclear antigen (PCNA) with Wt1 in epicardial cells, in interstitial mesenchymal cells or in the intramyocardial MSC clusters as early as 24 h after MI (C.K., unpublished). Endocardial proliferation and activation of subepicardial EMT, as well as mesenchymal transformation in cardiomyocyte progenitor cells under low oxygen tension, might also participate in the enrichment of the post-ischemic mesenchyme (Aisagbonhi et al., 2011; van Oorschot et al., 2011). Others reported that myofibroblasts derived from bone marrow could also make a significant contribution to enrichment of the mesenchyme, albeit with a considerable time delay (van Amerongen et al., 2008). However, evidence is limited for post-ischemic mobilization and migration of bone-marrow-derived MSCs to the heart (Hoogduijn et al., 2014). We did not detect any proliferation in existing cardiac myocytes in cardiac tissues in our study. Consequently, we think that epicardial EPO delivery following MI probably accelerates augmentation of the early proliferating intramyocardial mesenchymal cell mass that hosts proliferating cardiac MSCs where EMT signaling was induced. Studies are currently underway in our laboratory to track cardiac MSC origin, kinetics and fate in the ischemic heart.

TGF-β and WNT signaling are known to be essential niche factors regulating EMT in epicardial progenitors (as explained previously); these factors also have a role in regulating self-renewal, stemness and multipotency in MSCs (Gonzalez and Medici, 2014; Forte et al., 2012; Song et al., 2006; Nusse, 2008). In our study, focal analyses in intramyocardial cardiac MSC clusters documented activation of the EPO-R downstream signal mediator AKT in all EPO therapy groups in the peri-infarction area 24 h after MI. TGF-β signal expression and upstream signal mediator FOS were also upregulated in all EPO therapy groups in post-ischemic cardiac MSC clusters. Remarkably, we were able to demonstrate that WNT signaling was specifically triggered in cardiac MSC clusters following epicardial EPO delivery early after MI, as illustrated by increased intranuclear β-catenin and WNT receptor Fzd7 expression.

Moreover, we demonstrated early *Mmp2* and *Tgf-b* genetic upregulation in the ischemic heart after epicardial EPO delivery, which might have enhanced myofibrotic tissue reorganization by MSCs and other regenerative cells (van Wijk et al., 2012; van Oorschot et al., 2011; Dobaczewski et al., 2010; Nguyen et al., 2010). Importantly, we were able to successfully translate these results to human bone-marrow-derived MSCs. EPO stimulation of human MSCs resulted in immediate activation of the ERK/FOS axis, induction of the downstream target gene *TGF-B1*, increased TGF-β secretion and strong induction of canonical WNT signaling (with remarkably elevated *de novo* synthesis of ligand WNT-1 and WNT receptors *FZD1* and *FZD7*). Increased intracellular β-catenin content was also observed. These findings were all in accordance with previous reports (all signalings with reasonably respective time delays) (Danielyan et al., 2009; Chong et al., 2007). TGF-β secretion from human MSCs might have been predominately paracrine, because induced TGF-β signaling mediators SMAD2 and SMAD3 were not found to be activated in human MSCs at multiple time points within 24 h of EPO stimulation (C.K. and R.J., unpublished). Consequently, epicardial EPO delivery after MI might have primarily induced niche factors TGF-β and WNT signaling in intramyocardial cardiac MSC clusters, which secondarily paracrine-activated stromal fibroblasts and other cardiac stem cell niches promoting EMT and supporting intracardiac cell recruitment to the ischemic myocardium. It is noteworthy that we achieved a clear distinction of MSCs from vascular fibroblasts. In accordance with others, MSCs demonstrated lower WNT signaling inhibition, elevated levels of collagen and vimentin gene induction, and specific epigenetic collagen characteristics detected by RAMAN spectroscopy (Wagner et al., 2005; Movasaghia et al., 2007).

MSCs are known to be strongly involved in angiogenesis and vasculogenesis through direct endothelial and smooth muscle cell differentiation, as well as through paracrine activities (Gu et al., 2017). To the best of our knowledge, our results present for the first time a high angiogenetic potential of early post-ischemic cardiac CD45^−^CD44^+^DDR2^+^ MSCs. We demonstrated that the paracrine effect of cardiac CD45^−^CD44^+^DDR2^+^ MSCs on HUVECs was, at least in part, responsible for the induction of angiogenesis in HUVECs. Moreover, EPO stimulation synergistically increased the induced angiogenesis in addition to the paracrine activity from the cardiac CD45^−^CD44^+^DDR2^+^ MSCs. In addition, the EPC marker CD34 was found to increase very early after MI. This observation was in concordance with our previously illustrated EPC recruitment following effective EPO treatment in acute myocardial ischemia (Klopsch et al., 2009). Late after MI, improved angiogenesis and capillary density were exclusively found following epicardial EPO delivery. Consequently, these late improvements could have resulted from the early post-ischemic EPO-mediated mechanisms: (1) EPC recruitment, (2) proliferation, (3) TGF-β/WNT signaling in intramyocardial MSC niches, and (4) synergistic activation of angiogenesis with the cardiac CD45^−^CD44^+^DDR2^+^ MSCs.

Numerous studies have underlined the cardiomyogenic differentiation potential of cardiac progenitors (van Wijk et al., 2012; Zhou et al., 2008; Christoffels et al., 2009; Smart et al., 2011). Here, we provide the first evidence that continuous EPO stimulation could enhance the cardiomyogenic differentiation potential in cardiac CD45^−^CD44^+^DDR2^+^ MSCs, as shown by the increased expression of early transcription factor Nkx2.5. Zafiriou and colleagues reported that EPO increased proliferation in cardiac progenitors expressing myosin heavy chain during infarction healing (Zafiriou et al., 2014). Notably, our isolated cardiac MSCs partially revealed weak expression of early transcription factor GATA4. Myosin or actinin (specific for more mature cardiomyocytes) were not detected in mono-cultured cardiac MSCs, in co-cultured cardiac MSCs or in intramyocardial MSC clusters (C.K. and A.S., unpublished). The manuscript lacks a clear clarification of the fate of co-cultivated MSCs; however, the assessment time point was methodologically required because of a limited interval of stable co-cultivation. We have not seen a significant effect of applied MSCs on neonatal cardiomyocytes or on mature cardiac myocyte differentiation from cardiac CD45^−^CD44^+^DDR2^+^ MSCs. *In vivo* genetic cell-fate mapping in ischemic myocardial tissue will most probably be a more appropriate model to investigate these issues in the future. EPO-mediated promotion of immature cardiomyogenic differentiation in rat cardiac MSCs could not be translated to human MSCs (C.K., A.S. and H.L., unpublished). Instead, we demonstrated enhanced fibroblast differentiation in these bone-marrow-derived MSCs after continuous EPO stimulation, as detected by RAMAN spectroscopy. We, and others, reported tissue-specific differentiation potential, genetic programs and regenerative capacities in MSCs (Kwon et al., 2016; Gaebel et al., 2011a,b). With regards to signaling in erythropoiesis, EPO concordantly might have promoted tissue-specific differentiation and maturation in applied MSCs (Schnöder et al., 2015). Herein, we found clear EPO-mediated activations of AKT signaling and ERK signaling in MSCs, which are expected to interfere with multilinear differentiation (Song et al., 2006; Xu et al., 2007; Yang et al., 2005; Ward et al., 2007). Nevertheless, cardiac and bone-marrow-derived MSCs might primarily have participated in fibroblast generation, scar formation and myocardial fibrosis after MI (van Wijk et al., 2012; Crawford et al., 2012; Carlson et al., 2011). A more detailed study of subcellular signaling could tremendously improve our understanding of MSC cardiac-lineage differentiation capacity (Lemcke et al., 2017). Imaging for intra- and intercellular gene regulations, as well as respective cardiac-lineage transdifferentiation and reprogramming strategies, could be key factors that prospectively enhance the efficiency of stem-cell-based clinical trials whenever cardiac MSCs are targeted (Ieda et al., 2010; Qian et al., 2012; Jayawardena et al., 2012, Zangi et al., 2013; Muraoka et al., 2014; Hausburg et al., 2015; Lemcke et al., 2016).

In our study, epicardial EPO delivery resulted in superior left ventricular performance, reduced infarction size and attenuated cardiac remodeling after acute MI. Numerous studies have shown that early reduction of oxidative stress and myocardial tissue loss, early induction of angiogenesis and endothelial proliferation, AKT activation and mobilization of endothelial progenitors by EPO could initiate an improved MI healing process by limiting myocardial fibrosis and hypertrophy during late remodeling. We think that an early boost in regeneration by epicardial EPO delivery was the principal mechanism reducing pathologic remodeling, wall thinning of the IZ, infarction scaring and cardiomyocyte loss in our study. With regards to other studies, it is conceivable that angiogenesis and angiogenetic factors like EPO or vascular endothelial growth factor could directly (e.g. via AKT activation) and indirectly improve survival of cardiomyocytes, as well as preserve heart failure development, through later anti-fibrotic and anti-hypertrophic effects during MI healing and cardiac remodeling (Li et al., 2006, 2016; Klopsch et al., 2009; Nishiya et al., 2006; Gaebel et al., 2009; Mihov et al., 2009; Westenbrink et al., 2010). Disappointing clinical trials prompted us to investigate EPO-mediated regenerative mechanisms within the early time window of effective drug level (‘effective window’) after experimental MI (Stein and Ott, 2011). It was hoped that these studies, together with discussions of drug- and disease-dependent factors, could improve clinical results. Clinical MI primarily constitutes the end-stage of chronic coronary artery disease (CAD) in elderly patients. Numerous studies reported that myocardial hypoxia progressively activates remodeling (e.g. fibrosis, hypertrophy) and regenerative (e.g. stem cell recruitment, proliferation, angiogenesis, development of coronary collateral circulation) mechanisms before MI. Regenerative processes could, therefore, reduce myocardial vulnerability for acute anoxic damage (Koerselman et al., 2003). Note, regeneration initially started after immense anoxic damage and was not impeded by a chronic disease in our model of experimental MI. Kobayashi and Wang first introduced local high-dose EPO release from hydrogels for improved MI healing compared with conventional EPO delivery (Kobayashi et al., 2008; Wang et al., 2009). We, and others, had previously mentioned a possible dependency on EPO delivery, dosing and timing for effective regeneration (Prunier et al., 2007; Moon et al., 2005; Lipsic et al., 2004; Klopsch et al., 2009, Gäbel et al., 2009; Westenbrink et al., 2008; Zwezdaryk et al., 2007). Herein, we illustrated that even moderate EPO doses could activate early intracardiac regenerative mechanisms within the ‘effective window’, which might be strongly dependent on immediate post-ischemic epicardial EPO delivery and/or sustained intracardiac enrichment of EPO. In particular, induced proliferation and stem-cell niche regulation appeared relevant for regeneration and angiogenesis and were temporarily spatially attributed to the early intramyocardial post-ischemic mesenchyme and cardiac MSCs following epicardial EPO delivery. With regard to clinical MI scenarios, our experimental results raised new evidence that EPO might be most promising when administered locally and sustained as early as possible after large acute ischemic damage (e.g. ST elevation MI). Treatment might be particularly effective when applied to resident stem cell niches in patients with a short history of CAD. The therapeutic efficiency of EPO might be low in patients with a longer history of chronic CAD, however, because regenerative mechanisms might have been already activated for years before the onset of MI. In addition, chronic CAD and remodeling would progress after MI, thus decreasing regeneration capacity, functional recovery and late survival (Koerselman et al., 2003).

In conclusion, epicardial EPO delivery induced early intracardiac regenerative key mechanisms, which could be relevant for improved early MI healing, angiogenesis and enhanced late remodeling, capillary density, infarction size and cardiac performance. EPO-mediated early post-ischemic intracardiac enrichment of proliferating mesenchyme and TGF-β/WNT signaling in intramyocardial MSC niches most probably led to an early adequate supply of highly angiogenetic regenerative cells and boosted tissue transformation after MI. Focal analyses provide the first evidence that epicardial EPO administration might be the optimal route for early post-ischemic direct induction of TGF-β/WNT signaling through activation of AKT, upregulation of FOS and enhanced WNT receptor Fzd7 expression in intramyocardial cardiac MSCs. Of equal importance, EPO promoted strong synergistic angiogenesis with early post-ischemic cardiac CD45^−^CD44^+^DDR2^+^ MSCs from rats. Moreover, EPO could stimulate cardiomyogenic differentiation potential in cardiac CD45^−^CD44^+^DDR2^+^ MSCs. Our results were largely translated to human MSCs, which probably presented tissue-specific fibroblast differentiation potential driven forward by EPO. Regarding the necessity for progress in MI therapy, we emphasize that more consideration is required for the ‘effective window’ regarding temporarily, spatially and drug-delivery dependent mechanisms in acute MI healing. Further investigation is warranted for detailed analyses of the regeneration and differentiation capacities of cardiac post-ischemic MSCs as well as for subcellular EPO-mediated signaling.

## MATERIALS AND METHODS

The study conforms to the Declaration of Helsinki and human biomaterials were taken after informed written consent. Surgical and animal care protocols were reviewed and approved by the local animal care committees of Mecklenburg-Western Pomerania. For more detailed information on listed oligonucleotide primers, antibodies and selected gene arrays we refer to the ‘Supplementary information’.

### *In vivo* studies

### Experimental design

Lewis rats (*Rattus norvegicus*, male, 279±22 g, Charles River Laboratories) were randomly assigned to six groups. In the Sham operation group a left thoracotomy was executed (Sham, *n*=19). In two MI control groups rats received left anterior descending coronary artery ligation subsequently followed by systemic intraperitoneal and local intramyocardial saline control injections (MIC, *n*=25) or implantation of an epicardial fibrin patch (MIC-F, *n*=29). In all of the three therapy groups recombinant human EPO treatment (cumulative dose 300 U kg^−1^ bodyweight, Epoetin-α/Erypo^®^, Ortho Biotech, Division of Janssen Cilag GmbH, Germany) was delivered immediately after MI by systemic intraperitoneal (EPO-S, *n*=28) injection, local intramyocardial (EPO-L, *n*=26) injection or epicardially as an EPO-plus fibrin patch (EPO-F, *n*=29). A subset of randomly selected rats from all groups was sacrificed for hematological, biochemical, histological and real-time PCR evaluations at 24 h after coronary artery ligation. EPO pharmacokinetics were analyzed at multiple time points during the first 24 h. The remaining rats were assessed at 6 weeks for functional measurement and histological analysis.

### Generation of MI in rats and EPO application

Rats were anesthetized with sodium pentobarbital (Sigma-Aldrich, St Louis, USA; 50 mg kg^−1^, intraperitoneal administration) and the left anterior descending coronary artery (LAD) was permanently ligated 2 mm beyond its origin. Immediately after LAD ligation, all groups received intramyocardial injections of saline (MIC, MIC-F, EPO-S, EPO-F) or EPO (EPO-L). We aimed at a maximum intracardiac EPO tissue concentration of approximately 100 U g^−1^ (moderate dose level; estimated pre-operative rat heart weight 0.7 g). Each rat received three intramyocardial injections (25 µl each). Injections were given along the border of the blanched myocardium. Sham operated rats underwent identical surgical procedures without permanent LAD ligation, but followed by intramyocardial saline injections. The MIC and EPO-S rats received one intraperitoneal injection (500 µl) of either saline or 300 U kg^−1^ EPO, respectively. In groups MIC-F and EPO-F, a 200 µl fibrin hydrogel patch (containing 300 U kg^−1^ EPO in EPO-F or 0.9% saline in MIC-F) was implanted epicardially on top of the blanched myocardium and the surrounding border zone by local coagulation of 100 µl liquid human fibrinogen (10 mg ml^−1^) with 100 µl activating reagent containing CaCl_2_ and bovine thrombin. All components of the fibrin hydrogel patch were provided by RWTH Aachen University.

### Cardiac function late after MI

Six weeks after surgery, rats (Sham *n*=9, MIC *n*=8, EPO-S *n*=9, EPO-L *n*=9, MIC-F *n*=6, EPO-F *n*=6) underwent left ventricular catheterization obtaining pressure-volume (P/V) loops, as described previously (Klopsch et al., 2009). Briefly, data were collected with the Millar Pressure-Volume System [Ultra-Miniature Pressure-Volume Catheter (model SPR-838), Millar Pressure Conductance Unit (model MPVS300) and Millar PowerLab data acquisition hardware; emka Technologies, Paris, France]. P/V loops of the left ventricle (LV) were recorded under normal conditions (baseline) followed by stress conditions mediated by intravenous dobutamine administration (10 µg kg^−1^ min^−1^, Sigma-Aldrich). The animal was then allowed to rest for 30 min until the systemic hemodynamic condition was stable at baseline level, which was confirmed by permanent monitoring via conductance catheterization. Data were analyzed with IOX Version 1.8.3.20 software (emka Technologies).

### Infarction size and cardiac remodeling analyses late after MI

After P/V-loop measurements, rats were euthanized. Hearts (*n*=6 for each group) were removed, snap-frozen in liquid nitrogen and later sliced horizontally for histological analysis. Frozen transverse tissue sections (5 µm thick) from the mid-portion of the LV (base-to-apex axis) were stained with Fast Green FCF (Sigma-Aldrich) and Sirius Red (Division Chroma, Muenster, Germany). The infarction size (as a percentage) was determined by analyzing the epicardial length of the Sirius-Red-positive area, using the computerized planimetry (Axio Vision LE Rel. 4.5 software; Carl Zeiss, Jena, Germany), and relating it to the entire circumference of the LV. To determine the LV wall thickness (in µm) at the infarction area, five randomly selected measurements were performed inside the area that stained positive for Sirius Red in each tissue section (*n*=5 sections for each heart).

To evaluate collagen density and cardiomyocyte size inside the non-infarcted remote area (RA) of viable myocardium, LV transverse tissue sections (*n*=6, for each group) stained with Fast Green FCF and Sirius Red were analyzed using computerized planimetry. Sirius-Red-positive areas in the RA near the endocardial border were examined in ten randomly chosen fields per section at 200× magnification. Collagen density was expressed as the ratio of collagen area to myocardial area as a percentage. The size of Fast Green FCF positive cardiomyocytes in RA near the endocardial border was studied in parallel, employing 200× magnification as described previously (van Wijk et al., 2012). A total of 100 cardiomyocytes per heart were randomly chosen and measured by computerized planimetry (in µm^2^). Capillary density in the infarction area and peri-infarction border zone were evaluated using fluorescein-labeled *Lycopersicon esculentum* (Tomato) lectin (FL-1171; Vector Laboratories, USA) staining and analyzing ten randomly chosen fields per section at 400× magnification with computerized planimetry (in n mm^–2^).

### Hematological analyses and detection of EPO plasma and tissue levels

Venous blood was harvested either from the jugular vein at 10 min, 2 h and 8 h after EPO administration or from the right ventricle at 24 h after MI. Protein was extracted from interventricular septal myocardium applying the TRIZOL^®^ method at 24 h after MI. Reticulocyte count was examined by Sysmex XE-2100 (SYSMEX, Norderstedt, Germany). Plasma and myocardial tissue concentration of EPO were evaluated with enzyme-linked immunosorbant assay (ELISA) kits using Immulite Fa.DPC (Siemens, Bad Nauheim, Germany) and mouse anti-EPO monoclonal antibody (Euro/DPC, Gwynedd, UK). EPO concentrations were presented as mU ml^−1^ for plasma and mU g^−1^ protein for tissue (*n*=7 in EPO-F, *n*=8 in other groups).

### Quantitative real-time reverse transcription PCR of early intracardiac target genes

For analysis of mRNA levels, hearts (*n*=6, for each group) were removed 24 h after primary surgery and the LV was carefully dissected along the right ventricular free wall. The infarcted zone (IZ) including the peri-infarction penumbra zone from the LV was separated from the non-infarcted zone at the remote myocardium of the interventricular septum and snap-frozen in liquid nitrogen, as described previously (van Wijk et al., 2012). Total RNA was isolated following the instructions of the TRIZOL^®^ Reagent (Thermo Fisher Scientific, USA), including DNase treatment, and followed by reverse transcription of RNA for first strand cDNA synthesis (High Capacity cDNA Reverse Transcription Kit; Thermo Fisher Scientific). Samples were analyzed with NanoDrop1000 (Thermo Fisher Scientific) to demonstrate consistent quality and to determine RNA concentration. cDNA was then analyzed using quantitative real-time reverse transcription PCR in TaqMan gene expression assays. Primer sets for *in vivo* real-time PCR (Applied Biosystems) are summarized in Table S1. Amplification and detection were performed with the StepOnePlus™ Real-Time PCR System (Thermo Fisher Scientific) in TaqMan Universal Master Mix (Thermo Fisher Scientific) according to the instructions of the manufacturer (Thermo Fisher Scientific). cDNA extracts were tested in duplicate and negative controls included in each assay. Cycle thresholds (C_T_) for single reactions were determined with StepOne™ Software 2.0 (Applied Biosystems) and target genes were normalized against glyceraldehyde 3-phosphate dehydrogenase (GAPDH) (formula: ΔC_T_=C_T target_-C_T GAPDH_). ΔΔC_T_ were obtained using the Sham group as calibrator sample (formula: ΔΔC_T_=ΔC_T sample_-ΔC_T calibrator sample_). The 2^−ΔΔC^ method was employed to analyze the changes in gene expression after MI and different EPO treatments. The groups MIC and MIC-F were considered as not comparable at 24 h after MI (see Statistical analyses section).

### Intracardiac immunohistology early after MI

Primary antibodies for immunohistology are summarized in Table S2. Frozen transverse tissue sections (5 µm) from hearts were harvested 24 h after MI (*n*=6 for each group). Proliferation was detected and localized by immunostaining with primary antibody rabbit anti-PCNA followed by secondary antibody goat anti-rabbit Alexa Fluor 568 (Thermo Fisher Scientific, Carlsbad, USA) and counterstained with primary antibody mouse anti-vimentin followed by secondary antibody donkey anti-mouse Alexa Fluor 647 (Thermo Fisher Scientific). Vimentin-positive mesenchymal cell proliferation in the peri-infartion area was quantified in two tissue sections per animal [five randomly chosen high-power fields (HPF) at 400× magnification per section] and expressed as the ratio of (PCNA-positive+vimentin-positive cells) to the (total number of vimentin-positive cells) as a percentage.

To analyze the vimentin-positive mesenchymal cell density inside the peri-infarction zone, LV transverse tissue sections were stained for vimentin and analyzed using computerized planimetry. Vimentin-positive areas were examined in ten randomly chosen fields per section at 200× magnification. Mesenchymal cell density was expressed as the ratio of vimentin-positive area to myocardial area as a percentage.

For detection, localization and signal intensity analyses of TGF-β, FOS, intranuclear β-catenin, Fzd7 and pAKT, tissue sections were incubated with primary antibodies rabbit anti-TGF-β, rabbit anti-FOS, rabbit anti-β-catenin, rabbit anti-Fzd7 or rabbit anti-pAKT, respectively, followed by goat anti-rabbit Alexa Fluor 568 secondary antibody. Sections were counterstained with primary antibody mouse anti-vimentin followed by secondary antibody donkey anti-mouse Alexa Fluor 647 as well as with tomato lectin (FL-1171). Focal signal intensities were examined in between all groups in vimentin-positive MSC clusters (*n*=30 randomly selected clusters per signal in every MI group, *n*=10 clusters per signal in Sham), as described previously (Klopsch et al., 2017). Fluorescence signal intensity analyses were executed in close consistency with previous reports from our laboratory (Lemcke et al., 2017). Briefly, fluorescence signal intensity (Alexa Fluor 568) was quantified under standardized confocal laser scanning microscope settings (ELYRA PS.1 LSM 780, Carl Zeiss) and a relative fluorescence signal intensity was calculated after normalization against fluorescence from nuclear counterstaining (see below) as an internal control. ZEN 2011 software (blue edition, Carl Zeiss) was applied to obtain the fluorescence signal intensities.

In addition, intranuclear β-catenin signal expression from vimentin-positive mesenchymal cell clusters was compared with solitary interstitial mesenchymal cells, perivascular mesenchymal cells and cardiomyocytes in randomly chosen HPFs (400×).

Nuclei were counterstained with 4′,6-diamidino-2-phenylindole (DAPI, Thermo Fisher Scientific) in all sections. Matched isotype controls were performed for all stainings. Labeled sections were observed at 100-630× magnification on an ELYRA PS.1 LSM 780 confocal microscope (Carl Zeiss). The groups MIC and MIC-F were considered as not comparable at 24 h after MI (see Statistical analyses section).

### *In vitro* studies

### Experimental design

Rat early post-ischemic cardiac CD45^−^CD44^+^DDR2^+^ MSCs were isolated using fluorescence-activated cell sorting (FACS) of cardiac mononuclear cells (MNCs) from hearts at 24 h after MI. Cardiomyogenic differentiation of rat cardiac CD45^−^CD44^+^DDR2^+^ MSCs was tested in co-cultures with mouse neonatal cardiomyocytes regarding different EPO (100 U ml^−1^) conditioning times. The angiogenetic effect of rat cardiac CD45^−^CD44^+^DDR2^+^ MSCs on HUVECs was studied on Matrigel^®^ (Corning Inc., USA) under different culture conditions and additional EPO (100 U ml^−1^) stimulation.

In translational experiments standardized human bone marrow derived MSCs were analyzed using TaqMan real-time PCR assays, the ‘Human Mesenchymal Stem Cell PCR Array’ (SA Biosciences, USA), western blotting, ELISA and RAMAN spectroscopy regarding different EPO (100 U ml^−1^) conditioning times.

### Cell isolation of rat cardiac CD45^−^CD44^+^DDR2^+^ MSCs with fluorescence-activated cell sorting

Post-ischemic cardiac CD45^−^CD44^+^DDR2^+^ MSCs were isolated from infarcted (MIC) hearts 24 h after surgery, as described previously (Klopsch et al., 2017). Briefly, cardiac MNCs from ischemic hearts were first cultivated in Dulbecco's Modified Eagle Medium containing 10% fetal bovine serum (FBS) and 1% penicillin and streptomycin (DMEM-10, PAN-Biotech GmbH, Aidenbach, Germany) until reaching confluency. Thereafter, MNCs were incubated in the presence of fluorochrome-conjugated antibodies (CD45, CD44) and DDR2 primary antibody followed by incubation with fluorochrome-conjugated secondary antibody (Table S3). Cardiac CD45^−^CD44^+^DDR2^+^ cells were collected in DMEM-10 medium using BD FACS Aria II^®^ (BD Biosciences, Heidelberg, Germany). Re-analysis of the target CD45^−^CD44^+^DDR2^+^ MSCs was performed with FACS DIVA software (version 6.1.2; BD Biosciences). The target MSCs were further cultivated in DMEM-10 medium for a maximum of four passages. Medium was changed every 3 days.

### Cardiomyogenic differentiation of rat cardiac CD45^−^CD44^+^DDR2^+^ MSCs

Cardiomyogenic differentiation of rat cardiac CD45^−^CD44^+^DDR2^+^ MSCs was tested in co-cultures with mouse neonatal cardiomyocytes that were isolated as described previously (Lemcke et al., 2016). Briefly, hearts were extracted from neonatal Naval Medical Research Institute (NMRI) mice (1-2 days old, Institute for Experimental Surgery, Rostock, Germany), minced into small pieces (∼1 mm^3^), digested with an enzyme solution provided with Pierce™ Primary Cardiomyocyte Isolation Kit (Cat. No. 88281, Thermo Fisher Scientific) and further processed in accordance with the manufacturer's instructions. The obtained cell suspension was pre-plated on non-coated cell culture vessels for 90 min to allow the adherence of non-cardiomyocytes. Subsequently, the non-adherent cardiomyocyte cell fraction was collected and centrifuged at 300×***g*** for 10 min. Cardiomyocytes were cultured on gelatin-coated culture vessels (Sigma-Aldrich) and maintained in DMEM-10 medium.

Rat cardiac CD45^−^CD44^+^DDR2^+^ MSCs were labeled with Qtracker^®^ 565 Cell Labeling Kit (Thermo Fisher Scientific). The co-culture of rat MSCs with mouse neonatal cardiomyocytes in DMEM-10 medium was supplemented with EPO (100 U ml^−1^) and controlled against an unstimulated co-culture and an unstimulated MSC mono-culture regarding different EPO conditioning times (1 day or 7 days). Medium was changed every 2 days. Cardiac MSCs were co-cultured with neonatal cardiomyocytes at a ratio of 1:60. A monoculture of equal cardiac MSC number (2.5×10^4^) was correspondingly maintained. After 7 days cells were fixed with 4% formaldehyde and permeabilized in 0.2% Triton-X. Subsequently, primary antibodies against antigens for cardiomyogenic differentiation Nkx 2.5 and GATA4 were tested. Antibodies are summarized in Table S2. For visualization, fluorochrome-conjugated secondary antibodies donkey anti-goat Alexa Fluor 488 and donkey anti-rat Alexa Fluor 647 were added, respectively. Cells were counterstained with DAPI (Sigma-Aldrich). Relative fluorescence signal intensities of intranuclear Nkx2.5 and GATA4 in Qtracker^®^-labeled cardiac MSCs were quantified under standardized confocal laser scanning microscope settings (ELYRA PS.1 LSM 780, Carl Zeiss) and application of ZEN 2011 software (blue edition, Carl Zeiss). MSC identification was affected by cellular overlay of MSCs and cardiomyocytes in the co-culture. For clear MSC identification we performed a careful examination for definitive cytosolic Qtracker^®^ expression and a nuclear cell diameter >10 µm. The mean fluorescence intensities of MSCs from co-cultures were normalized against respective fluorescence intensities of MSCs from mono-cultures in at least 50 HPFs (400×) per culture group.

### Angiogenetic potential of rat cardiac CD45^−^CD44^+^DDR2^+^ MSCs

HUVECs were isolated from human umbilical cords harvested post-partum, as described previously (Gaebel et al., 2011a,b). Thereafter, HUVECs were cultivated in endothelial growth medium (EGM-2; Lonza, USA) at 37°C in a humified atmosphere containing 5% CO_2_. HUVECs (passages 1-3) were centrifuged at 300 ***g*** for 10 min at 37°C before experiments. For the angiogenesis assay, 24-well plates were coated with 300 µl Matrigel^®^ (4°C, Growth Factor Reduced Basement Membrane Matrix, Corning Inc.) and incubated for 30 min at 37°C. Subsequently, 35,000 HUVECs/well in DMEM-10 basal medium were plated on Matrigel^®^-coated wells (*n*=4 well per group). Angiogenesis was examined qualitatively for the formation of endothelial tubuli and networks after 24 h, employing phase-contrast microscopy (Carl Zeiss Axiovert 40 CFL) and ELYRA PS.1 LSM 780 confocal microscope (Carl Zeiss). The number of cellular branches per HPF (100×) and the number of cellular junctions per HPF (100×) were analyzed quantitatively by computerized planimetry with ImageJ Software (angiogenesis analysis module, National Institutes of Health, USA) in six randomly chosen fields per well. Investigations were performed with regard to five different sets of culture conditions: (1) HUVEC control group with HUVECs in DMEM-10 basal medium; (2) HUVECs+MSCs group with HUVECs in co-culture with rat cardiac CD45^−^CD44^+^DDR2^+^ MSCs; (3) Cond. DMEM-10 group with HUVECs in conditioned DMEM-10 medium (cultivated with rat cardiac CD45^−^CD44^+^DDR2^+^ MSCs for 48 h); (4) DMEM-10+EPO group with HUVECs in DMEM-10 supplemented with EPO (100 U ml^−1^); and (5) Cond. DMEM-10+EPO group with HUVECs in conditioned DMEM-10 medium (cultivated with rat cardiac CD45^−^CD44^+^DDR2^+^ MSCs for 48 h) supplemented with EPO (100 U ml^−1^).

### Human relevance analyses in translational *in vitro* experiments with human MSCs

Bone marrow samples were obtained by sternal aspiration from patients (median age 70 years) undergoing coronary artery bypass graft surgery at Rostock University Medical Center. Human MSC isolation and culture in standard Mesenchymal Stem Cell Growth Medium (MSCGM™, Lonza) were performed as described previously (Gaebel et al., 2011a,b). Cells were harvested when reaching 70-80% confluency and stored in liquid nitrogen. After the third passage, MSCs were used for subsequent *in vitro* experiments. Immunophenotypic characterization and multi-lineage differentiation capacity has been illustrated elsewhere (Gaebel et al., 2011a,b). For experiments, MSCs were cultivated in DMEM-10 supplemented with EPO (100 U ml^−1^; DMEM+EPO group) and controlled against a DMEM control group (DMEM-10 without EPO supplementation) and a standard MSCGM control group. Medium was changed three times per week. EPO effect on MSCs was investigated by plating MSCs differentially at 150,000 cells per well on 12-well plates for short-term analyses with western blot (EPO conditioning times 10 min, 30 min, 60 min, 24 h; *n*=6 wells per time point and group), at 80,000 cells per well on 24-well plates for short-term analyses in quantitative real-time PCR (EPO conditioning times 1 h, 6 h, 24 h, *n*=4 wells per time point and group) and enzyme-linked immunosorbant assay (EPO conditioning time 24 h, *n*=6 wells per group) and at 50,000 cells per well on 6-well plates for PCR array and RAMAN spectroscopy (14 days cultivation, *n*=6 wells per group).

Human umbilical artery fibroblasts (Moreira et al., 2014) were purified from umbilical cords and cultured in DMEM-10. Fibroblasts served as a reference cell source.

### Real-time reverse transcription PCR in human MSCs

RNA isolation, cDNA synthesis, amplification and detection were performed as explained earlier for the *in vivo* PCR studies (see above). Total RNA was isolated from fibroblasts and cultured MSCs separately for all wells. cDNA was analyzed using quantitative real-time reverse transcription PCR in TaqMan gene expression assays (selectively for *TGF-B1*, *FOS*, *SMAD2*, *SMAD3*, *WNT1*, *FZD1* and *FZD7*) and a PCR array. Fibroblasts were exclusively examined with the PCR array.

TaqMan gene expression assays for quantitative real-time PCR were obtained from Thermo Fisher Scientific and are summarized in Table S1. C_T_ of target genes were normalized against the ribosomal protein *RPLP0* housekeeping gene (formula: ΔC_T_=C_T target_-C_T RPLP0_). Resulting ΔC_T_ of duplicates was averaged and ΔΔC_T_ were obtained using the standard culture setting in the MSCGM control group as calibrator sample (formula: ΔΔC_T_=ΔC_T sample_-ΔC_T calibrator sample_). In the current study, the 2^−ΔΔCt^ method was employed to analyze the changes in gene expression in the DMEM control group or DMEM+EPO group.

For the PCR array, analysis samples were exclusively pooled. Obtained from MSC well plates, 50 ng of cDNA per well were pooled separately for all groups. Accordingly, 300 ng total cDNA was employed from cultured fibroblasts. Gene induction profiles were analyzed using the ‘Human Mesenchymal Stem Cell PCR Array’ (PAHS-082c, SA Biosciences, USA) for selected molecules (see Table S4), including the housekeeping gene *GAPDH* and negative controls according to the manufacturer’s protocol and the RT² qPCR mastermix (SA Biosciences). The ‘RT² Profiler PCR Array Data Analysis Template’ v4.0 (SA Biosciences) was used to analyze PCR data. The target genes were normalized against the *GAPDH* housekeeping gene (formula: ΔC_T_=C_T target_–C_T GAPDH_). Resulting ΔC_T_ were shown for groups MSCGM control, DMEM control, DMEM+EPO and the fibroblast reference.

### Western blotting in human MSCs

MSCs and fibroblasts were harvested by medium aspiration and boiled in lysis buffer [2% sodium dodecyl sulfate (SDS), 10% glycerol, 5 mmol l^−1^ ethylenediaminetetraacetic acid (pH 8.0), 62.5 mmol l^−1^ Tris-HCl (pH 6.8), 0.01% 3,3′,5,5′-tetrabromophenolsulfonphthalein, 5% β-mercaptoethanol] for 5 min. Subsequently, samples were centrifuged at 13,000×***g*** for 5 min and stored at −20°C until analysis. Western blotting was performed as described previously (Lange et al., 2012). Briefly, cellular proteins were separated by 8% SDS polyacrylamide gel electrophoresis, blotted onto PVDF membrane and sequentially probed with primary antibodies against phosphorylated ERK (pERK), ERK, pAKT, AKT, phosphorylated β-catenin (pβ-catenin), β-catenin and GAPDH (see Table S2). IRDye^®^ 800CW and IRDye^®^ 680CW conjugated secondary antibodies were applied for detection of primary antibody binding. Using an Odyssey^®^ Infrared Imaging System, all immunoblots were scanned at a wavelength of 700 nm for detecting IRDye^®^ 680-labeled antibodies and at a wavelength of 800 nm for IRDye^®^ 800CW-conjugated antibodies. The content of target protein was quantified by means of the Odyssey^®^ software version 3.16. GAPDH was used as a protein loading control. Values for pERK, pAKT and pβ-catenin protein were normalized to the total ERK, AKT and β-catenin protein contents, respectively.

To compare the intracellular β-catenin protein content between groups (MSCGM control, DMEM control and DMEM+EPO), values were normalized against housekeeping protein GAPDH.

Results obtained from the MSCGM control group were set to 100% as calibrator sample and employed to present changes in the DMEM control group, DMEM+EPO group and the fibroblast reference (see Statistical analyses section).

### Enzyme-linked immunosorbant assay of secreted TGF-β in human MSCs

The effect of EPO on MSCs was investigated after an EPO conditioning time of 24 h (*n*=6 wells per group). Supernatants of MSC cultures were received from the DMEM control group and DMEM+EPO group after 24 h cultivation. Levels of secreted TGF-β1 were quantified using an ELISA Ready-Set-Go Kit (88-8350-22, eBioscience) according to the manufacturer's protocol. The detection sensitivity limit was 8 pg ml^−1^ for TGF-β1. Equal volumes of blank culture medium served as negative control.

### RAMAN spectroscopy in human MSCs

After finishing MSC culture, 200 cells per well (*n*=6 wells) were harvested, pooled separately for each MSC culture setting and fixed in 4% formaldehyde. Accordingly, 1200 arterial fibroblasts underwent identical processing. For RAMAN spectroscopy, at least 40 cells from each cell culture setting were randomly selected per analysis. The BioRam^®^ system (CellTool GmbH, Germany; 785 nm laser, 80 mW, 60-fold water immersion objective) was employed, scattering cross-sections for excitation wave numbers between 760-3000 cm^−1^. Applying the particular component (PC) analysis and the NIPALS algorithm, wave numbers have been investigated that can dissociate analyzed cell types and be assigned to biological characteristics (Movasaghia et al., 2007). Spectroscopy analyses were repeated three times single-blind. Data analysis was performed with The Unscrambler X, Version 10.3 (CAMO Software AS, Norway).

### Statistical analyses

Statistical analyses were performed using Sigma Stat software version 3.5 (SPSS Inc., Chicago, USA). Results are expressed as mean±s.d. Results in the figures are expressed as mean±s.e. of the mean (s.e.m.). Overall comparisons of the treatment groups were performed as far as appropriate by using the one-way analyses of variance (ANOVA) method that applies post-hoc multiple Holm-Sidak tests, and by using the nonparametric Kruskal-Wallis (failing normality) or post-hoc multiple Dunn tests. *P* values <0.05 were considered statistically significant.

For *in vitro* studies, changes between rat cardiac MSCs from mono-cultures and co-cultures were analyzed statistically under consideration of different culture conditions (medium conditions, EPO incubation times). With regard to the results obtained from human biomaterial, the group MSCGM control was set to 100%. Respective changes in gene and protein expression were calculated in the DMEM control group, in the DMEM+EPO group and in the fibroblast reference. Changes between the DMEM control group and DMEM+EPO group were analyzed statistically under consideration of different EPO incubation times. The fibroblast reference was analyzed statistically against the DMEM control group and MSCGM control group.

For *in vivo* studies, overall comparability between groups was limited. The molecular effects of fibrin in the epicardial patch were not controlled against a fibrin-free three-dimensional epicardial patch. Therefore, early changes in gene and protein expression were considered as not comparable in the groups MIC and MIC-F at 24 h after MI (except for EPO pharmacokinetics). As a consequence, three comparisons have been executed: (1) *in vivo* results obtained at 24 h after MI in the groups EPO-S and EPO-L were exclusively compared with MIC; (2) *in vivo* results obtained at 24 h after MI in the group EPO-F were exclusively compared with MIC-F and (3) at 6 weeks after MI overall comparison between groups was performed to assess and compare therapeutic efficiency, as well as to identify final myocardial tissue changes following different EPO treatment strategies. However, comparability might still have been limited.

## Supplementary Material

Supplementary information
