# Supplementary material for: Intramyocardial angiogenetic stem cells and epicardial erythropoietin save the acute ischemic heart
Source: Dis Model Mech. 2018 Jun 22;11(6):dmm033282. doi: 10.1242/dmm.033282 (PMC6031356; doi:10.1242/dmm.033282)
Supplement: Supplementary information [file dmm-11-033282-s1.pdf]

Table S1 - List of primers used in real time PCR

Primer	Catalog Number
EPO-R (rat)	Rn00566533_m1
TNF-α (rat)	Rn00562055_m1
Bcl-2 (rat)	Rn999999125_m1
Cyclin D1 (rat)	Rn00432359_m1
Cdc2a (rat)	Rn00570728_m1
SDF-1 (rat)	Rn00573260_m1
CXCR4 (rat)	Rn00573522_s1
CD34 (rat)	Rn02102859_s1
TGF-β1 (rat)	Rn01418715_m1
MMP 2 (rat)	Rn01538175_m1
GAPDH (rat)	Rn99999916_s1
FOS (human)	Hs99999140_m1
SMAD2 (human)	Hs00183425_m1
SMAD3 (human)	Hs00969210_m1
WNT-1 (human)	Hs01011247_m1
Fzd1 (human)	Hs00268943_s1
Fzd7 (human)	Hs00275833_s1
TGF-β (human)	Hs00998133_m1
RPLP0 (human)	Hs99999902_m1

Table S2 - List of antibodies used in Western blotting and Immunostaining.

Antibody	Application	Dilution	Host	Manufacturer (Catalog Number)
TGF-β	IHC	1:200	Rabbit	Abcam (ab66043)
Vimentin	IHC	1:300	Mouse	Abcam (ab8069)
Lectin	IHC	1:50	Lycopersicon Esculentum	Vector Laboratories (FL-1176)
PCNA	IHC	1:300	Rabbit	Cell Signaling (#13110)
CD29	IHC	1:100	Rabbit	Abcam (ab52971)
CD44	IHC	1:50	Rabbit	Abcam (ab24504)
CD90	IHC	1:50	Goat	Santa Cruz Biotechnology (sc-6071)
CD105	IHC	1:100	Rabbit	Abcam (ab107595)
PDGFRα	IHC	1:50	Rabbit	Cell Signaling (#3164)
DDR2	IHC	1:20	Rabbit	Bioss (bs-4194R)
β-Catenin	IHC, WB	1:1000	Rabbit	Cell Signaling (#9562)
FOS	IHC	1:50	Rabbit	Abcam (ab64636)
Fzd7	IHC	1:50	Rabbit	Abcam (ab190289)
pβ-Catenin	WB	1:1000	Rabbit	Cell Signaling (#9561)
ERK	WB	1:5000	Rabbit	Millipore (#06-182)
pERK	WB	1:1000	Rabbit	Cell Signaling (#9101)
AKT	WB	1:1000	Rabbit	Cell Signaling (#4691)
pAKT	IHC, WB	1:100, 1:1000	Rabbit	Cell Signaling (#4060)
GAPDH	WB	1:5000	Rabbit	Cell Signaling (#2118)
Nkx 2.5	IHC	1:100	Goat	Santa Cruz Biotechnology (sc-8697)
GATA4	IHC	1:100	Rat	eBioscience (#14-9980-80)

Table S3- List of antibodies for flow cytometry and FACS.

Primary / secondary Antibody	Conjugate	Dilution	Host	Manufacturer (Catalog Number)
CD29	Fluorescein, FITC	1:100	Armenian hamster	eBioscience (11-0291)
CD44H	Phycoerythrin, PE	1:333	Mouse	eBioscience (12 – 0444)
CD90	-	1:555	Mouse	BioLegend (202510)
CD105	-	1:100	Mouse	Millipore (#05-1424)
PDGFRα	-	1:200	Rabbit	Cell Signaling (#3164)
DDR2	-	1:50	Rabbit	Bioss (bs-4194R)
CD45	Allophycocyanin, APC	1:50	Mouse	eBioscience (17-0461)
secondary anti-mouse	Alexa Fluor 488	1:300	donkey	Thermo Fisher Scientific (A21202)
secondary anti-rabbit	Alexa Fluor 488	1:300	donkey	Thermo Fisher Scientific (A21206)
streptavidin	PE	1:222	-	BioLegend (405204)

Disease Models & Mechanisms • Supplementary information

Table S4 - Gene selection from "Human Mesenchymal Stem Cell PCR Array"

Abbreviation	Molecule
BMP2	Bone morphogenetic protein 2
CD44	Cluster of differentiation 44
COL1A1	Collagen 1 α 1
CTNNB1	Catenin β 1
FGF2	Fibroblast growth factor 2
GCF15	Granulocyte colony-stimulating factor 15
HGF	Hepatocyte growth factor
ICAM1	Intercellular adhesion molecule 1
IL1B1	Interleukin 1 β 1
ITGA6	Integrin α 6
ITGAV	Integrin α 5
ITGB1	Integrin β 1
KITLG	Kit-ligand, stem cell factor (=CD 117)
MMP2	Matrix metalloproteinase 2
NOTCH1	Notch type-1 transmembrane proteine family
PDGFRB	Platelet-derived growth factor receptor β
RUNX2	Runt-related transcription factor 2
SMAD4	Common-mediator SMAD regulating signal transcription
TGFB3	Transforming growth factor β 3
THY1	Thymocyte antigen 1 (=CD 90)
VCAM1	Vascular cell adhesion molecule 1
VEGFa	Vascular endothelial growth factor a
VIM	Vimentin
VWF	Von Willebrand factor

Disease Models & Mechanisms • Supplementary information
